# Electrostatic Tailoring of Freestanding Polymeric Films for Multifunctional Thermoelectrics, Hydrogels, and Actuators

**DOI:** 10.1021/acsnano.4c12502

**Published:** 2024-12-09

**Authors:** Suo Tu, Ting Tian, Jinsheng Zhang, Suzhe Liang, Guangjiu Pan, Xiaoxin Ma, Liangzhen Liu, Roland A. Fischer, Peter Müller-Buschbaum

**Affiliations:** † Technical University of Munich, TUM School of Natural Sciences, Department of Physics, Chair for Functional Materials, James-Franck-Str. 1, 85748 Garching, Germany; ‡ Chair of Inorganic and Metal−Organic Chemistry, Department of Chemistry and Catalysis Research Center (CRC), TUM School of Natural Sciences, Technical University of Munich, 85748 Garching, Germany

**Keywords:** PEDOT:PSS films, electrostatic self-assembly, thermoelectrics, hydrogels, actuators

## Abstract

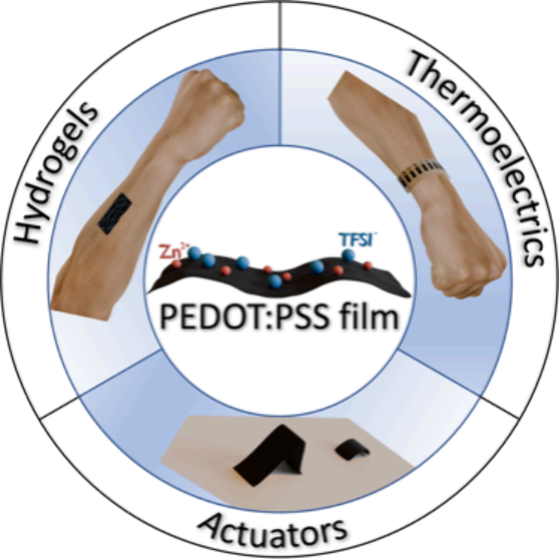

Organic conducting polymer poly(3,4-ethylenedioxythiophene):poly(4-styrenesulfonate) (PEDOT:PSS) has garnered enormous attention in organic electronics due to its low-cost solution processability, highly tunable conductivity, superior mechanical flexibility, and good biocompatibility together with excellent atmospheric stability. Nevertheless, limited electrical properties and unfavorable water instability of pristine PEDOT:PSS film impede its further implementation in a broad spectrum of practical applications. In this work, the successful tailoring of the intrinsic electrostatic interaction within PEDOT:PSS and consequent optimized electrical properties are enabled by a simple yet effective ionic salt post-treatment strategy. The choice of zinc di[bis(trifluoromethylsulfonyl)imide] (Zn(TFSI)_2_) not only endows the post-treated PEDOT:PSS film with high electrical properties but also other compelling characteristics, including superior water stability, excellent mechanical flexibility, and fast humidity responsiveness. Multidimensional characterizations are conducted to gain in-depth insights into the mechanisms underlying such improved performance, ranging from intermolecular interactions, polymer conformations, and doping levels to microstructural characteristics. Benefiting from these versatile properties, the as-prepared freestanding Zn(TFSI)_2_-post-treated PEDOT:PSS films can serve as promising candidates for high-performance polymeric materials integrated into multifunctional flexible electronics, including thermoelectric power generators, conductive hydrogels, and humidity-responsive actuators. This study demonstrates a facile methodology for the exploration of multifunctional conducting polymers, whose implications can extend across a wide range of next-generation wearable devices, bioelectronics, and soft robotics.

## Introduction

Recent advances in organic semiconductor engineering have offered more opportunities for wearable technology, bioelectronics, and soft robotics capable of interacting well with the human body and adapting to dynamic environments.^1−4^ Among existing organic semiconductors, π-conjugated conducting polymers, such as poly(3,4-ethylenedioxythiophene) (PEDOT), polyacetylene, polyaniline, polypyrrole, and polythiophene, can provide a versatile and reliable platform for such technology owing to the attractive merits of inherent softness, flexibility, and conductivity.^5,6^ In contrast to their traditional and rigid inorganic counterparts, these soft conductors could also mimic biological organisms and are enabled with good compliance and adaptiveness, thereby achieving next-level functionalities and broadening the application range.^7,8^ Despite substantial research efforts in organic electronics thus far, integrating a single soft conductor into multiple technological applications remains less explored, predominantly due to the unavoidable trade-offs and grand challenges of fulfilling all requirements.

Particularly, poly(3,4-ethylenedioxythiophene):poly(4-styrenesulfonate) (PEDOT:PSS) has emerged as one of the most promising representatives owing to its high tunability in ion/electronic conductivity, mechanical properties, aqueous dispersibility, solution processability, and manufacturing compatibility.^9^ By meticulous tailoring, the PEDOT:PSS can realize a combination of properties highly desirable for diverse applications, covering actuation, active sensing, and energy harvesting.^10−13^ However, developing a simple all-in-one methodology for effective tailoring of PEDOT:PSS toward multifunction targeted applications is imperative yet challenging. Notably, addressing the issue of poor electrical properties is one of the critical prerequisites for PEDOT:PSS-based electronic devices to deliver satisfying performances. For example, with extensive research dedicated to high power factors in the field of thermoelectrics (TEs), simultaneous improvements in conductivity (σ) and Seebeck coefficient (*S*) have long been sought for PEDOT:PSS.^14,15^ Due to a competitive relationship in between, the strategies effective in enhancing σ often compromise *S*, and vice versa.^16−18^ For example, Dong et al. reported an alkali-metal-ion base treatment, resulting in an improvement in the *S* at a great sacrifice of the σ of the resultant PEDOT:PSS film.^19^ As a commercially available polyelectrolyte, the intrinsically conducting PEDOT is surrounded by excess electrically insulating PSS via electrostatic interactions in an aqueous medium.^14,15,20,21^ Following water evaporation and PEDOT film formation, the PEDOT moieties responsible for hole transport are still embedded within a hygroscopic, insulting PSS matrix, thus leading to poor electrical properties.^20^ To overcome such intrinsic limitations, the most frequently adopted approaches primarily focus on doping, dedoping, post-treatment, or a combination of these to facilitate PSS removal and/or regulate the PEDOT oxidization level.^15^ Essentially, all these existing methods can be interpreted as delicate control of the electrostatic self-assembly of PEDOT so that the advantageous characteristics of straight chain conformation, high-level crystalline ordering, and fibril-like nanomorphology are achieved simultaneously.

For integration into the emerging technology of hydrogel bioelectronics, PEDOT:PSS polymer networks are required to offer tissue-mimetic mechanical properties as well as high conductivity.^22^ Unlike biological tissues that are intrinsically water-rich and soft, the pristine, dry, and high-Young’s modulus PEDOT:PSS needs further physical and mechanical engineering to be suitable for practical applications.^11,12^ The incorporation of nonconductive polymers to form interpenetrating PEDOT hydrogels can serve as an effective strategy to improve the mechanical properties, which, however, unavoidably sacrifices electrical conductivity.^23^ On the other hand, introducing inorganic conductive filler can significantly improve the electrical conductivity but can lead to potential compromises in stability and biocompatibility.^11,12,22^ More critically, non-cross-linked yet hygroscopic PSS chains that exist in excess make PEDOT:PSS susceptible to structural disintegration upon water exposure,^24,25^ thus limiting its practical use as a bioelectronic interfacing material in wet physiological environments.

Significant progress has recently been achieved for soft robotics systems integrating PEDOT:PSS-based actuators by leveraging ubiquitous physical mechanisms to generate deformation.^10,26−29^ Stemming from the sensitive hygroscopic nature of PSS, PEDOT:PSS can function as an ideal humidity-responsive actuator, and tremendous efforts have been devoted to improving the actuation speed, mechanical robustness, and energy-dissipating capability.^10,26^ Analogously, the desirable characteristics are mutually exclusive, and realizing one favorable performance parameter is often traded off against another one or two. Thus, urgent demand exists for a combination of desirable properties for PEDOT:PSS to meet all criteria required by a specific target application. However, breaking off multiple compromises has been persistently challenging and has rarely been realized so far. In this regard, integrating such a rigorous set of merits demanded by a multifunctional thermoelectrics/hydrogel/actuator system would substantially complicate the design principles and narrow the processing window of PEDOT:PSS.

The seminal work of Ghosh et al. demonstrated the ionic cross-linking of PEDOT:PSS with the help of MgSO_4_, resulting in an optimal conductivity approaching 1 S cm^–1^.^30^ Later on, the successful construction of PEDOT:PSS hydrogels was enabled in the presence of ionic salts.^31,32^ All these previous reports suggested a favorable impact of ionic salts on modifying PEDOT-based conducting polymers. In the present work, we explore a facile one-step post-treatment strategy using three ionic salts, consisting of identical bis(trifluoromethylsulfonyl)imide (TFSI) anion yet varied (1-ethyl-3-methylimidazolium bis(trifluoromethylsulfonyl)imide (EMIM)/lithium (Li)/zinc (Zn) cations, to regulate the electrostatic interactions of PEDOT:PSS via a favorable ionic exchange reaction. Unlike the direct mixing of the PEDOT:PSS ink with ionic salts reported in previous work,^33^ ionic salts are used to post-treat PEDOT:PSS films considering that an aggregated solution would impede the following film deposition. All ionic salt species can yield simultaneous optimizations in the Seebeck coefficient and conductivity, with the best performance parameters achieved by Zn(TFSI)_2_. To the best of our knowledge, relevant works using Zn(TFSI)_2_ post-treatment to obtain freestanding PEDOT:PSS films have not been reported so far. Although multistep pretreatment or/and post-treatment combinations can generally further boost the electrical properties, most of these methods no longer highlight or study the improvement of the cross-linking level of the polymer networks but rather overcome the trade-off between σ and *S* via multistep optimizations. This work highlights the development of high-performance freestanding PEDOT:PSS films via a simple one-step ionic salt post-treatment for multifunctional applications. The multiple-length scale structures from the molecular configuration to the long-range conductive network elucidate a simultaneous increase in *S* and σ. As verified by systematic spectroscopic and structural investigations, controlling the binding strength of ionic salts is effective in accessing positive deviations from trade-off relations and thus bestowing PEDOT:PSS with a wide range of advantageous characteristics. Benefiting from the partial removal of hydrophilic PSS chains and a high cross-linking level of the hydrophobic PEDOT polymer network, the Zn(TFSI)_2_-post-treated PEDOT:PSS film is also endowed with good mechanical robustness, with structural collapse being stabilized upon direct water immersion, which makes highly conductive PEDOT:PSS films particularly suitable for conductive hydrogel and actuator applications since PEDOT:PSS films remain robust during the working service. Given a difference in swellability of the soluble PSS domains and the insoluble PEDOT domains at the nanoscale level, the resulting robust PEDOT:PSS films are expected to exhibit a humidity-responsive actuation behavior. With most existing works focusing on thin films with thicknesses typically ranging from 10 nm to 1 μm, we fabricate the thicker freestanding PEDOT:PSS layers with the objective of seamless merging with electronic devices. As a proof-of-concept demonstration, the Zn(TFSI)_2_-post-treated PEDOT:PSS is selected as a model system and used as an active layer for further integration in multifunctional applications. Consequently, the Zn(TFSI)_2_-post-treated PEDOT:PSS enables wearable TE generators with enhanced TE output power, conductive hydrogels with high conductivity and electrical self-healing ability, and single-layer actuators with good moisture responsiveness. Overall, we establish a close loop relationship of multiple-length scale structure, versatile properties and multifunctional applications. This work provides multidimensional mechanistic understandings of ionic salt-induced performance improvements of PEDOT:PSS, which is also expected to offer insights and more opportunities for the development of next-generation multifunctional applications in wearable organic electronics, bioelectronics, and soft robotics.

## Results and Discussion

### Modulation of Electrostatic Self-Assembly

**Figure 1:**
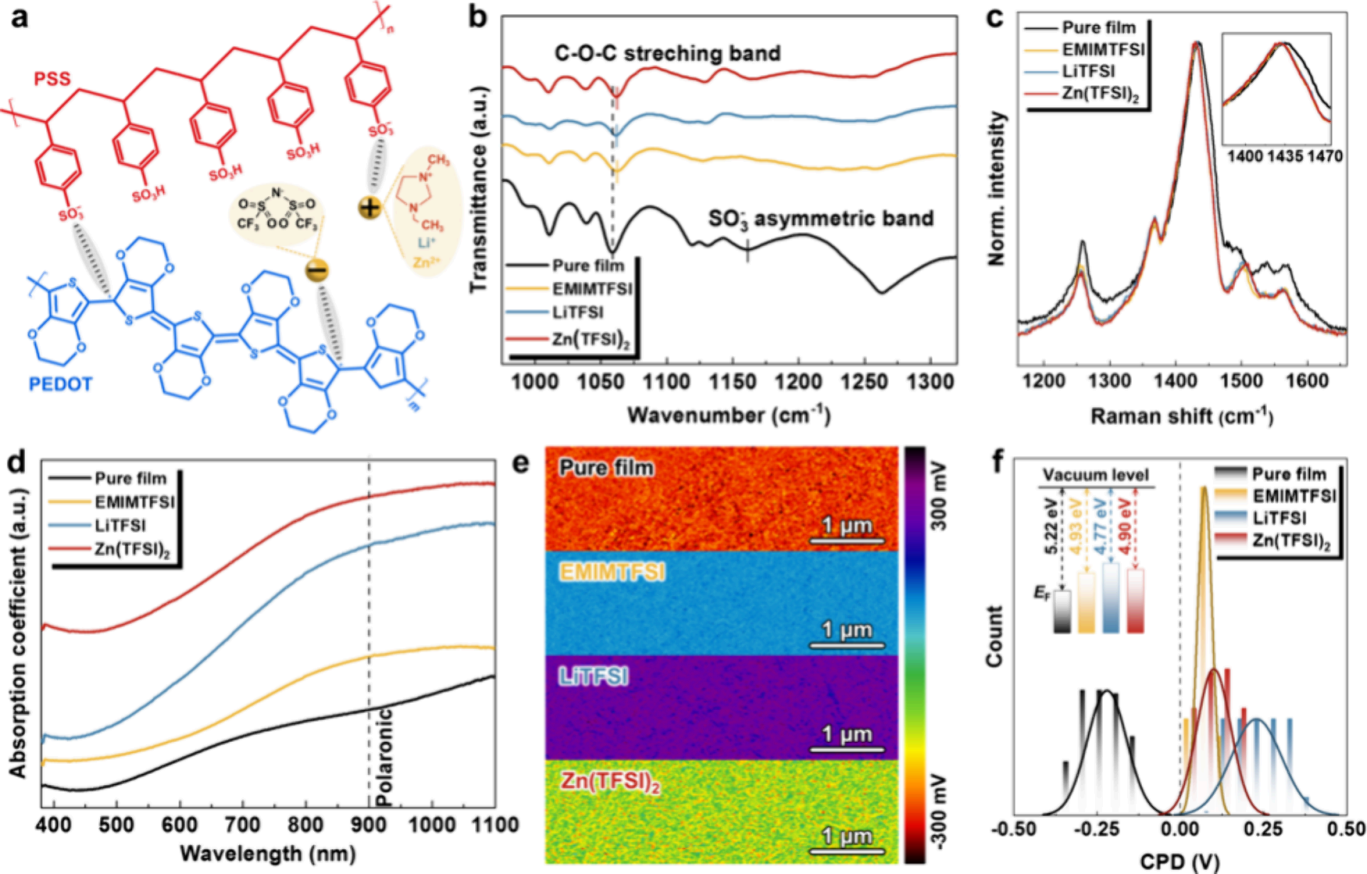
Changes in intermolecular interactions, polymer conformations, and doping levels of PEDOT:PSS films post-treated with ionic salts. (a) Chemical structures of the semiconducting polymer PEDOT:PSS and three different types of salts. (b) FTIR spectra, (c) Raman spectra, and (d) UV–vis-NIR absorption of PEDOT:PSS with or without the salt post-treatments. (e) KPFM images and (f) corresponding surface potential distribution of PEDOT:PSS with or without the salt post-treatments.

Figure 1a presents the chemical structures of the semiconducting polymer PEDOT:PSS that is electrostatically connected, along with selected ionic salts of three different categories. These salts have TFSI^–^ anions in common, while the cations vary from EMIM^+^ to Li^+^ to Zn^2+^. Within the pristine PEDOT:PSS sample, the positively charged PEDOT moieties typically interact with oppositely charged PSS moieties due to a preferential electrostatic binding capability. To monitor the electrostatic interaction and ion exchange between PEDOT:PSS and ionic salts on a molecular level, Fourier transform infrared (FTIR) spectroscopy is used (Figure 1b). Characteristic of the C–O–C stretching vibration of the PEDOT moiety, the distinct band located at 1059 cm^–1^ in the pure film shifts to higher wavenumbers upon all salt post-treatments. This observation indicates the presence of TFSI^–^ anions as the counterions of PEDOT^+^ cations. In addition, the vibrational fingerprint for PSS (1161 cm^–1^SO_3^−^_ Such an elimination of the degeneracy in the SO_3^−^_ asymmetric vibration further confirms the ion exchange process.^34^ Corresponding to the C_α_-C_α′_ inter-ring,^19^ the peak at 1260 cm^–1^ gets weakened upon salt post-treatment. Analogous to the previous report,^35^ this is attributed to the decrease in the polarization caused by the ion-pair formation of PEDOT^+^ with TFSI^−^.^36,37^ The peak at 1520 cm^–1^ belonging to the symmetric C_α_=C_β_ stretching of the thiophene ring^19^ is not detectable for all samples (Figure S1), indicating that PEDOT segments still remain at a high oxidation level even upon salt post-treatment. To verify the possible chemical dedoping of PEDOT:PSS upon salt post-treatment, we further perform Raman spectroscopy analysis using an excitation wavelength of 532 nm (Figure 1c). The most prominent symmetric C_α_=C_β_ stretching vibration of PEDOT (between 1400 and 1450 cm^–1^) in the pristine film becomes narrower and more intense after the salt post-treatment. This signifies a decreased contribution of the oxidized PEDOT, as the reduced segments of PEDOT are more active at such a green excitation.^13,38−40^ With salt post-treatment, this characteristic signal can also be identified to shift toward lower wavenumbers, implying a more linear conformation of PEDOT is induced.^41^ In general, this conformational transformation from benzoid to quinoid structure results in increased charge-carrier mobility and thus leads to enhanced conductivity in the film. Besides, the shoulder at 1497 cm^–1^, corresponding to the asymmetric C_α_=C_β_ in-plane stretching vibration of PEDOT chains, gets stronger and shifts to higher wavenumbers after the salt post-treatments. This finding is characteristic of the increased benzoid population in PEDOT chains, as reported in the literature.^42−44^ To gain further insights into the chemical doping level of PEDOT chains, UV–vis-NIR spectra are used (Figure 1d). Typically, PEDOT chains exist in three oxidation levels known as neutral, polaron, and bipolaron states.^45^ Compared to the pure film, the emergence of a polaronic absorption band at ca. 900 nm can be identified in the salt-post-treated samples. Such a discrepancy is indicative of the decreased population of bipolarons due to a partial reduction of PEDOT.^46,47^ In addition, Kelvin probe force microscopy (KPFM) measurements reveal the salt-induced alteration of the electronic structure in PEDOT:PSS, as suggested by the different surface potentials shown in Figure 1e. By extracting the contact potential difference (V_CPD_) between the tip and the sample,^48^ the work function (WF) is determined. Being smaller than the 5.22 eV in the pure PEDOT:PSS film, the mean WF values are 4.93, 4.77, and 4.90 eV for EMIMTFSI-, LiTFSI-, and Zn(TFSI)_2_-post-treated films, respectively (Figure 1f). Such a salt-induced WF reduction can be attributed to the PSS removal (Figure S2) and the above-mentioned dedoping effect, consistent with previous reports.^38,49^

Thus, we conclude that salt post-treatment can affect the oxidation degree of the PEDOT chains and the conformation of PEDOT:PSS. Regarding the detailed mechanisms underlying the salt post-treatments, they can be primarily rationalized from the following two aspects: (i) Specifically, the salt post-treatment loosens the PSS chain wrapped around PEDOT, enabling PEDOT molecules to selectively separate from insulating PSS chains and thus ultimately achieving high planarity. As such, a more extended conjugated length and a higher ordering level of PEDOT stacking favor the formation of conducting channels. (ii) As a consequence of ionic exchange, the Coulomb attraction between PEDOT and PSS is weakened. This gives rise to an increase in the polaron density and a decrease in the oxidation level, resulting in localized charge carriers on the backbone.^50^ Due to such synesthetic effects, a simultaneous improvement in electrical conductivity and the Seebeck coefficient is expected to be achieved in the salt-post-treated PEDOT:PSS films.

### Thermoelectric Properties and Mechanical Flexibility

**Figure 2:**
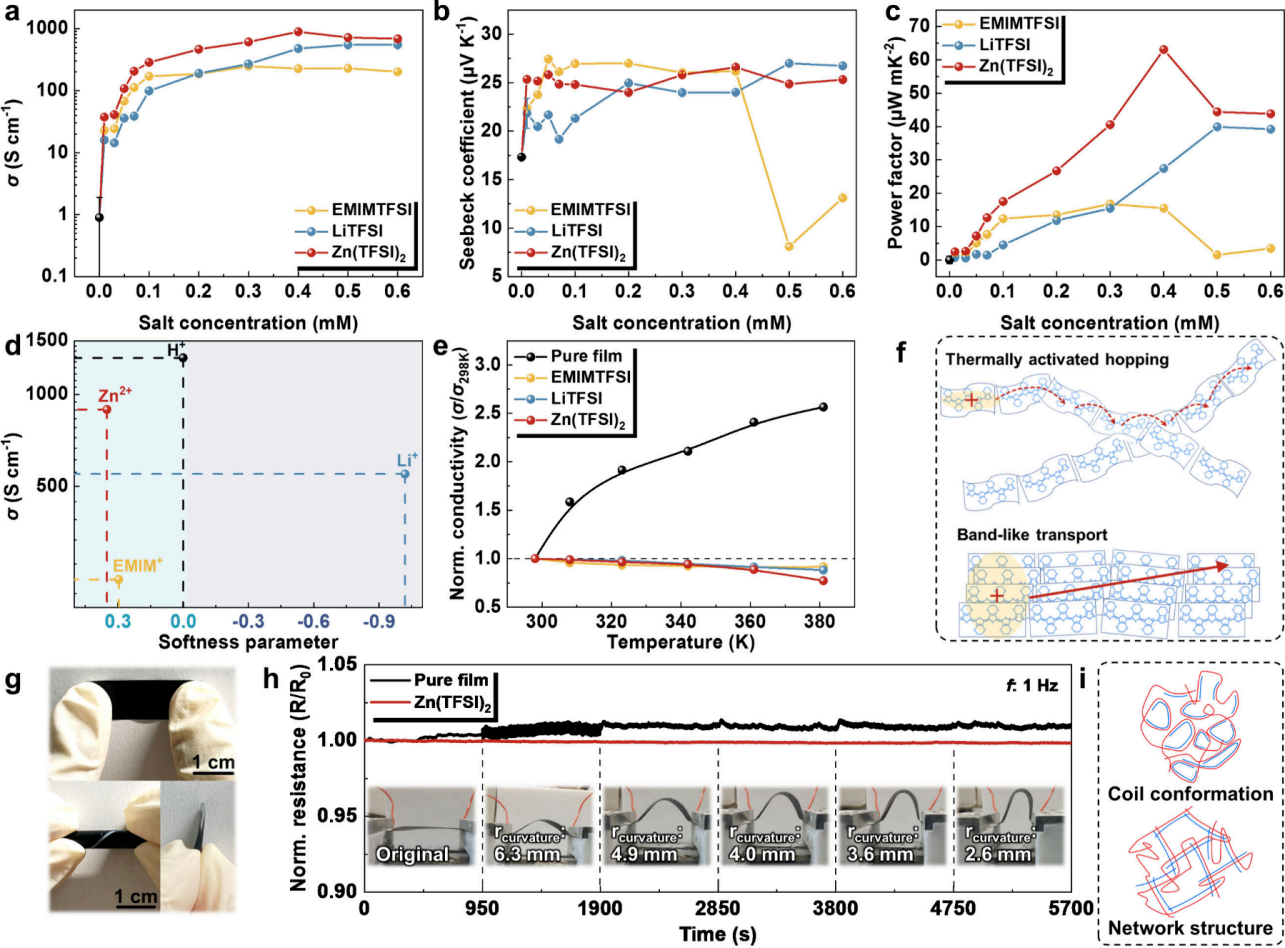
Thermoelectric and mechanical properties of PEDOT:PSS films post-treated with ionic salts. Thermoelectric properties of PEDOT:PSS films after post-treatment with different concentrations of salt solution at room temperature: (a) electrical conductivity, (b) Seebeck coefficient, and (c) power factor. (d) Relationship between the softness parameter of cations and the electrical conductivity of PEDOT:PSS films post-treated with the optimal salt concentration. (e) Normalized electrical conductivity of different PEDOT:PSS films as a function of temperature. (f) Electronic charge transport mechanism: thermally activated hopping transport of a relatively localized electronic charge carrier and band-like transport of a relatively delocalized electronic charge carrier. (g) Digital photographs of Zn(TFSI)_2_-post-treated PEDOT:PSS film in its original, wrapped, and folded states. (h) The normalized resistance of PEDOT:PSS film before and after Zn(TFSI)_2_-post-treatment as a function of curvature radii in the range of 2.6–6.3 mm. The inset shows the corresponding device photographs after bending using different curvature radii. (i) Schematic diagram of the conformation of PEDOT:PSS film before and after Zn(TFSI)_2_-post-treatment.

To validate these anticipated effects, we first compare the in-plane thermoelectric properties of all salt-post-treated PEDOT:PSS films at varied salt concentrations, as illustrated in Figure 2a–c. The σ of the pure PEDOT:PSS film is measured to be ca. 0.9 S cm^–1^, which is comparable to that reported in the literature.^51^ With poor charge carrier transport, this low conductivity is predominantly due to the excessive presence of insulating PSS species.^13^ Irrespective of the salt categories, a monotonously increasing trend of σ with increasing salt concentration is demonstrated until reaching a platform, as shown in Figure 2a. Notably, a small amount of salt for post-treatment can dramatically improve the σ by 2–3 orders of magnitude. Despite being distinctly different, the maximum σ achieved for each salt-post-treated PEDOT:PSS series is within a concentration range of 0.3–0.5 mM. The maximum σ that can be obtained follows an increasing trend in the order of EMIMTFSI-post-treated (228.8 S cm^–1^), LiTFSI-post-treated (548.3 S cm^–1^), and Zn(TFSI)_2_-post-treated (892.4 S cm^–1^) PEDOT:PSS films. We do not increase the concentration further, as more undesirable residues within the film would inevitably degrade σ (Figure S3). As shown in Figure 2b, the obtained positive *S* of the pure PEDOT:PSS film is 17.3 μV K^–1^, in agreement with the values in previous reports.^52,53^ Similar to σ, the *S* of all post-treated PEDOT:PSS films follows a fast rise with the salt concentration, and the maximum *S* slightly fluctuates within a narrow range of ca. 24–27 μV K^–1^. When the salt concentration is over 0.5 mM, an abrupt drop of *S* is noticeable in the case of the EMIMTFSI-post-treated films. Being hydrophobic and water-immiscible, EMIMTFSI residuals remain within the film even after water washing compared with the other two hydrophilic salts (LiTFSI and Zn(TFSI)_2_) (Figure S3). This detrimental effect can be attributed to the salt residuals, and a similar trend can be found for unrinsed Zn(TFSI)_2_-post-treated PEDOT film (Figure S4). Thus, the salt post-treatments can indeed give rise to a simultaneous enhancement of *S* and σ. Correspondingly, an optimized power factor of 16.8, 39.9, and 63.1 μW mK^–2^ is obtained for the PEDOT:PSS film post-treated with EMIMTFSI, LiTFSI, and Zn(TFSI)_2_, respectively (Figure 2c). Compared to previous investigations, the TE properties obtained here are still relatively impressive (Figure S5 and Table S1). Thus, the salt post-treatment gives rise to the simultaneous enhancement of *S* and σ. As a function of charge carrier concentration, an anticorrelation between *S* and σ has been well established (typically, σ increases whereas *S* decreases with charge carrier concentration). *S* is charge-carrier-mobility-independent, whereas σ is charge-carrier-mobility-dependent. Considering these dependencies, the simultaneous increased *S* and σ values in post-treated samples evidence the improvement of the charge carrier mobility induced by a high proportion of quinoid structure, which outcompetes the reduction in charge carrier concentration generated by dedoping.

The different levels of enhancement of σ in post-treated PEDOT:PSS films are mainly attributable to the cations in ionic salts, as they share identical anions. The cationic effect of metal chlorides on the PEDOT:PSS film cannot be simply interpreted by the valence state, as shown in Figure S6. In contrast, the softness parameters calculated for these metal cations coincide with the trend found in the electrical conductivity of corresponding post-treated films to a greater extent. The use of such a softness parameter of the cation to quantify the interactions between ionic salts and PEDOT:PSS has also been documented in previous literature.^54^Figure 2d summarizes the relationship between conductivity and ionic salts by evaluating the softness parameter of the cations.^53,55−57^ Typically, Zn^2+^ and EMIM^+^ salt with a positive softness parameter are expected to exert a more significant effect on the improvement of σ than Li^+^ salt with a negative softness parameter, as the former has a higher binding capability with PEDOT:PSS.^56^ This trend is further evidenced by a more evident aggregation of PEDOT:PSS solution upon EMIMTFSI and Zn(TFSI)_2_ incorporation than LiTFSI incorporation (Figure S7). However, EMIM^+^ yields a lower improvement of σ than Li^+^, predominantly due to the hydrophobic and water-immiscible properties of EMIMTFSI.^58^ As donated by H^+^ with a softness parameter of 0 in Figure 2d, the σ obtained by common but harshly concentrated H_2_SO_4_ post-treatment reaches 1313.9 S cm^–1^. The obtained σ of salt-post-treated PEDOT:PSS in this work is still competitive with the results achieved by many other complex engineering methods.^59^

As shown in Figure 2e, a positive and negative temperature dependence of σ can be found for the pure and post-treated PEDOT:PSS films, respectively. The opposite trend suggests that the salt post-treatment transforms the primary charge carrier transport from a hopping-like semiconductor into a band-like metallic conductor (Figure 2f).^60,61^ Due to a lack of π-electronic density overlapping between adjacent PEDOT chains, thermally activated hopping dominates the hole transport in the pure PEDOT:PSS film. By decreasing such a disorder, the PEDOT chains with more quinoid structures form after salt post-treatments, which leads to band-like hole transport and increased charge-carrier mobility. With the increased average temperature, the increase in the corresponding Seebeck coefficients also supports this band-like charge-carrier transport mechanism (Figure S8).

In addition to improved electrical properties, the as-prepared Zn(TFSI)_2_-post-treated PEDOT:PSS film exhibits enhanced flexibility. As demonstrated in Figure 2g, this freestanding strip can wrap around a rod with a radius of 2.5 mm in an intact way and be easily folded. To quantitatively evaluate the excellent flexibility, the resistance change (R/R_0_) upon bending is examined as a function of the radius of curvature. In contrast to pure film, almost negligible fluctuations in R/R_0_ and *S*/*S*_0_ can be found for the Zn(TFSI)_2_-post-treated PEDOT:PSS film during bending tests, indicating its high mechanical endurance (Figures 2h and S9). Furthermore, the resistance output is independent of the bending rate (Figure S10). Thus, the PEDOT:PSS film can act as a flexible and stretchable strain sensor by monitoring the ΔR/R changes upon deformation movements (Figure S11). Due to a strong Coulomb attraction between PEDOT and PSS, the pure film favors the formation of coil-like structures.^62^ Such a disordered morphological feature is unfavorable for further polymer chain rearrangements under stress (Figure 2i). By contrast, the salt post-treatments can weaken the Coulomb attraction through ion exchange and facilitate the generation of continuous network structures in an expanded coil-like conformation. Such an interconnected framework is beneficial for stress relaxation by polymer chain slippage,^34^ thus contributing to the enhanced mechanical properties of Zn(TFSI)_2_-post-treated PEDOT:PSS film.

### Microstructure Analysis

**Figure 3:**
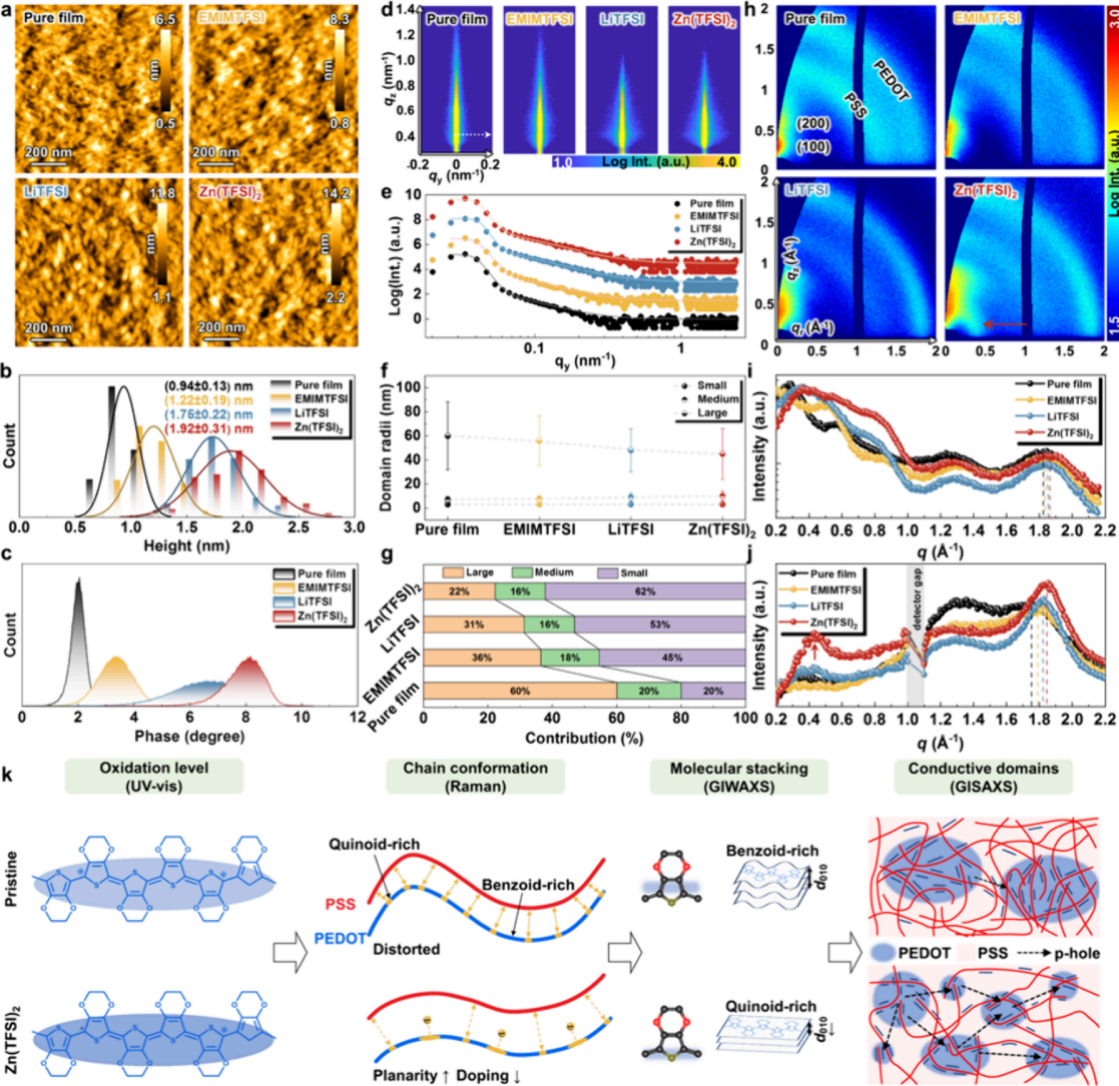
Microstructure analysis of PEDOT:PSS films post-treated with ionic salts. (a) AFM height images, (b) root-mean-square surface roughness distributions, (c) phase distributions of PEDOT:PSS films. (d) 2D GISAXS data of PEDOT:PSS films. (e) Horizontal line cuts (filled circles) with respective modeling results (solid lines) of PEDOT:PSS films. For clarification, all curves are shifted along the vertical axis. (f) Characteristic domain sizes of small-, medium-, and large-sized structures extracted from the GISAXS modeling results. (g) Quantity distribution of small-, medium-, and large-sized structures. (h) 2D GIWAXS data of PEDOT:PSS films prepared by various salt post-treatments. 1D profiles obtained from radial cake cuts of the 2D GIWAXS data along the (i) out-of-plane directions and (j) in-plane directions. (k) Schematic diagram of multilength scale structure of the pristine and Zn(TFSI)_2_-post-treated PEDOT:PSS films.

To provide a direct evidence about the salt-induced morphological alterations mentioned above, atomic force microscopy (AFM) is used to visualize the phase separation. After the salt post-treatments, grain aggregations with more pronounced grain boundaries are clearly visible, indicative of the formation of PEDOT-rich regions. (Figure 3a). Moreover, the root-mean-square (RMS) roughness increases from 0.94 nm for the pure film to 1.22, 1.75, and 1.92 nm for the PEDOT:PSS film post-treated with EMIMTFSI, LiTFSI, and Zn(TFSI)_2_, respectively (Figure 3b). Associated with the polymer conformational changes mentioned above, the surface roughness variations upon salt post-treatments are analogous to previous reports.^63^ Correspondingly, the bright PEDOT-rich and dark PSS-rich domains can be more easily distinguished from the phase images (Figure S12).^64^ Moreover, all salt-post-treated PEDOT:PSS films show significantly right-shifted and more broad phase distribution than the pure film (Figure 3c), signifying thinner insulating PSS-rich barriers surrounding conductive PEDOT-rich phases. These morphological observations collectively indicate that more prominent phase separation occurs on the film surfaces after the salt post-treatments. Although PEDOT-rich and PSS-rich domains are phase-separated, no significant differences can be distinguished from these elemental mappings (Figure S13).

In addition to surface structural arrangements, the importance of inner morphology on the conductivity of the PEDOT:PSS films was also underscored in our previous investigations.^46,65−68^ For this reason, grazing-incidence small-angle X-ray scattering (GISAXS) measurements are further performed to track the evolution in structural dimensions and spatial arrangements within the film. As shown in the 2D GISAXS data (Figure 3d), distinct scattering intensity distributions along the in-plane and out-of-plane directions represent different packing modes of PEDOT:PSS within these films. Notably, ionic salt post-treatment causes a much stronger lateral scattering observed from all post-treated films. As indicated by the white dotted arrow in Figure 3d, the horizontal line cuts are extracted from the material-sensitive Yoneda region of PEDOT. To access the lateral phase separation quantitatively, successful GISAXS data modeling is performed using three characteristic cylindrical structures, as plotted in Figure 3e. The large radii of PEDOT domains have a value of >45 nm; medium radii of PEDOT domains are ca. 7–10 nm, and small radii of PEDOT domains are ca. 3 nm, as summarized in Figure 3f. The large-sized PEDOT domains decrease from pure film (60 ± 28 nm) to EMIMTFSI-post-treated film (56 ± 21 nm), LiTFSI-post-treated film (48 ± 18 nm), and finally to Zn(TFSI)_2_-post-treated film (45 ± 21 nm). However, the medium-sized PEDOT domains increase from pure film (7.0 ± 2.4 nm) to EMIMTFSI-post-treated film (7.5 ± 2.6 nm), LiTFSI-post-treated film (9.0 ± 3.4 nm), and finally to Zn(TFSI)_2_-post-treated film (10.0 ± 4.0 nm). These structural parameters closely match the previous investigations.^66−68^ At the sacrifice of the contraction of large PEDOT-rich domains, the generated fine and densely distributed small PEDOT domains play a critical role in the overall interdomain electrical conductivity within PEDOT:PSS films. To provide more morphological insights, the extracted domain size distributions are illustrated in Figure 3g. Upon ionic salt post-treatments, the large- and medium-sized PEDOT domains decrease in quantity, whereas the small-sized domains increase significantly in number. Such a relative contribution confirms that salt post-treatment promotes the disintegration of the large- and medium-sized PEDOT domains to generate finer and denser-distributed small-sized PEDOT domains. Therefore, the salt-induced beneficial effect on electrical conductivity is also verified from the perspective of nanoscale morphology.

Figure 3h shows the grazing incidence wide-angle X-ray scattering (GIWAXS) data, providing detailed information about the arrangements of PEDOT:PSS on a molecular level. The 1D radial cake cuts performed along out-of-plane and in-plane directions are compared in Figure 3i and 3j, respectively. The pronounced peaks at *q* < 1 Å^–1^ correspond to alternately stacked PEDOT lamellae, and the broad halos centered at ca. 1.3 Å^–1^ and 1.8 Å^–1^ are characteristic of randomly arranged PSS chains and *π–π* stacking of crystallized PEDOT chains, respectively.^69^ According to Bragg’s law (*d* = 2π/*q*), the lattice *d*-spacing can be determined by momentum transfer *q*. With the (h00) peaks shifting to higher *q* regions (Figure 3i), the smaller *d* spacing indicates the formation of more compact PEDOT lamellae after the salt post-treatments. As such, the smaller *d*_(h00)_ spacing facilitates the beneficial charge carrier transport inside the crystal.^50,70^ Besides, the characteristic (010) scattering also shifts toward the high *q* positions with salt treatments. Since charge carrier transport along the *π–π* stacking direction is the rate-determining step,^71^ a slight contraction of *d*_(010)_ spacing can significantly enhance overall electrical conductivity by effectively improving interchain charge transfer. In both out-of-plane and in-plane directions, the (010) Bragg peak shifts toward higher *q* positions in the order of pure film < EMIMTFSI-post-treated PEDOT:PSS film < LiTFSI-post-treated PEDOT:PSS film < Zn(TFSI)_2_-post-treated PEDOT:PSS film, which is in good agreement with the electrical conductivity result. Besides, the increased edge-on orientation (Figure S14) that contributes to the charge carrier transport parallel to the substrate surface further confirms high σ. The presence of a distinct (100) peak in the in-plane direction (indicated by the red arrow in Figure 3h and 3j) of the Zn(TFSI)_2_-post-treated PEDOT:PSS film probably suggests that the cation of the Zn(TFSI)_2_ salt has the most significant effect on the σ of the PEDOT:PSS film compared with the other two salts. Combining systematic comparisons discussed above, Figure 3k schematically summarizes the multilength scale structure changes in the Zn(TFSI)_2_-post-treated PEDOT:PSS film achieved via electrostatic interaction control. With multidimensional manipulation ranging from doping level, chain conformation, crystalline ordering, and conductive domain connectivity, the TE trade-off relation is overcome, thus facilitating simultaneous enhancement of σ and *S*.

### Multifunctional Thermoelectrics, Hydrogels, and Actuators

**Figure 4:**
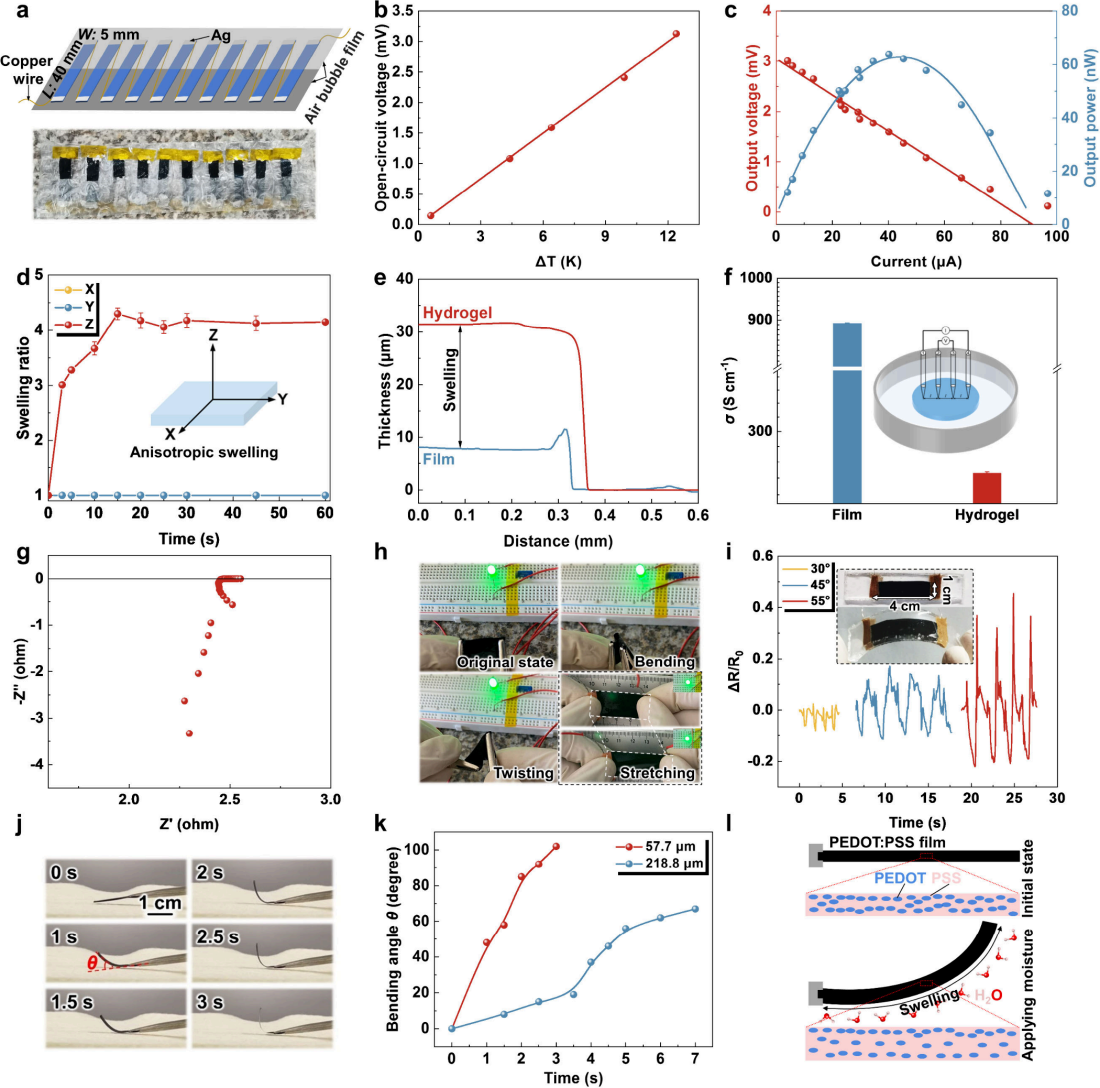
Thermoelectric power generators, conductive hydrogels, and humidity-responsive actuators of the PEDOT:PSS-based films. (a) Schematic illustration of the homemade flexible TE generator module composed of as-fabricated Zn(TFSI)_2_-post-treated PEDOT:PSS films with the air bubble film. (b) The open-circuit voltage as a function of temperature difference. (c) Output thermovoltage and output power versus current at a temperature difference of 12.6 K. (d) Anisotropy of dimension changes during the water swelling of Zn(TFSI)_2_-post-treated PEDOT:PSS hydrogel (X: width; Y: length; Z: thickness). (e) Thickness comparison between Zn(TFSI)_2_-post-treated PEDOT:PSS film and corresponding hydrogel. The presence of the peak in the blue line is caused by the debris of the scratched film during the thickness determination. (f) Conductivity comparison between Zn(TFSI)_2_-post-treated PEDOT:PSS film and hydrogel. The inset shows the schematic of the conductivity measurement for the Zn(TFSI)_2_-post-treated PEDOT:PSS hydrogel. (g) Nyquist plot of Zn(TFSI)_2_-post-treated PEDOT:PSS hydrogel. (h) Digital photographs of Zn(TFSI)_2_-post-treated PEDOT:PSS hydrogel connected to an LED bulb with hydrogel in different deformation modes. (i) Resistance change of Zn(TFSI)_2_-post-treated PEDOT:PSS hydrogel upon human finger bending. The inset shows photographs of PDMS-encapsulated Zn(TFSI)_2_-post-treated PEDOT:PSS hydrogel for a strain sensor. (j) Digital photographs of the Zn(TFSI)_2_-post-treated PEDOT:PSS strip (*l* × *w* × *h*: 20 mm × 3 mm × 57.7 μm), showing time-dependent moisture response. (k) Bending angle variations of Zn(TFSI)_2_-post-treated PEDOT:PSS strips (*l* × *w*: 20 mm × 3 mm) of two different thicknesses as a function of placement time under the same external humidity stimuli. (l) Schematic diagram of humidity-responsive mechanism in a single-layer PEDOT:PSS-based actuator.

Having demonstrated the capability of ionic salts in effective modulation of the PEDOT:PSS films from multidimensional structure–property characterizations, the Zn(TFSI)_2_-post-treated PEDOT:PSS film with optimal TE performance is selected as the model system to explore the application range that can be expanded. First, we fabricate flexible TE generator modules using the Zn(TFSI)_2_-post-treated PEDOT:PSS films to evaluate their performance in thermal energy harvesting and power generation. Figure 4a displays the geometry of the as-designed TE device, in which ten legs constituted by Zn(TFSI)_2_-post-treated PEDOT:PSS film with a dimension of 40 mm × 5 mm are electrically connected in series by silver paste and copper wire. Interspersed inside and outside the air bubble film, these legs are also thermally connected in parallel. With temperature differences (Δ*T*) applied, the open-circuit voltage outputs provided by the as-fabricated TE device demonstrate a linear correlation, as plotted in Figure 4b. In response to a Δ*T* increasing from ca. 0.6 to 12.4 K, the thermovoltage generated is linearly improved from ca. 0.15 to 3.13 mV. As exemplified by the current dependence at a fixed Δ*T* of 12.6 K, the output voltage and power of the obtained TE device follow a linear and parabolic-like correlation, respectively (Figure 4c). When the value of the loading resistance is close to that of the internal resistance of the TE device (Figure S15), the obtained output power reaches the maximum level of ca. 63 nW (Figure 4c). Such a favorable thermovoltage generation capability can ensure effective heat-to-electricity conversion, and thus, will be advantageous for practical TE applications. As a proof of concept, such TE modules are tested to demonstrate their potential in harvesting the common heat resource ubiquitous in daily life. Leveraging the biothermal between human body temperature and the ambient environment, the TE modules worn on a human wrist generate an output voltage of ca. 0.96 mV (Figure S16a), which is quite close to the critical minimum required to power wearable electronics (ca. 1 mV).^72^ Also, such a flexible TE device can be adhered closely to the curved surface of a beaker (Figure S16b). When the poured ca. 75 °C water reaches halfway through the TE modules (ca. 200 mL), a voltage difference of ca. 5.6 mV is rapidly produced (Figure S16b and Movie S1). Considering that the ionic dissociation of the sodium hydroxide (NaOH) in an aqueous medium is exothermic,^73^ the higher amounts of NaOH pellets added into a fixed volume of water can generate higher temperature gradients. By monitoring the evolution of the generated thermovoltage by the TE modules, the time scale associated with the complete dissolution of different molars of NaOH pellets can be quantified. As distinguished by distinct decay curves shown in Figure S17, the time required for 0.1, 0.3, and 0.5 molar of NaOH to dissolve in ca. 200 mL water is ca. 7, 12, and 15 h, respectively.

Next, we demonstrate a facile fabrication of the conductive hydrogel by one-step swelling of the Zn(TFSI)_2_-post-treated PEDOT:PSS film and its potential application for wearable electronics in strain sensing. In contrast to the disintegration observed in pure PEDOT:PSS film, the Zn(TFSI)_2_-post-treated PEDOT:PSS film remains intact even after being soaked in water for 24 h (Movie S2 and Figure S18). Such high water stability in the Zn(TFSI)_2_-post-treated PEDOT:PSS film can be attributed to a high cross-linking level of PEDOT, which also constitutes one of the determinants for the effective conversion into the conducting hydrogel.^22^Figure 4d shows the swelling behavior of Zn(TFSI)_2_-post-treated PEDOT:PSS film along the X, Y, and Z coordinate axes as a function of the soaking time. Typical of anisotropic swelling, no traceable changes occur along the X and Y directions with time. In stark contrast, the swelling ratio over the thickness direction (Z) follows a quickly increasing tendency within the first 15 s and then stabilizes at a platform afterward. Not surprisingly, this swelling anisotropy is inherited from the anisotropic solvent evaporation typical of film fabrication, in which the underlying substrate constitutes a mechanical constraint and hinders the PEDOT precursor ink from isotropic drying. Upon the film-to-hydrogel transition of Zn(TFSI)_2_-post-treated PEDOT:PSS, as shown in Figure 4e, the thickness substantially increases from ca. 7.7 μm to ca. 31.7 μm. Likewise, the noticeably enhanced H_2_O-related characteristic band in the FTIR spectra strongly evidence the presence of an appreciable amount of water in the hydrogel (Figure S19).^74^ As visualized from the cross-sectional SEM image (Figure S20), the freeze-dried hydrogel is abundant in porous skeletons, characteristic of the structural changes induced by the water swelling. Besides, a multilayered stacking framework can reduce mechanical damage during bending,^26^ thus facilitating improved long-term mechanical stability. Such a favorable microstructural feature also contributes to the high mechanical endurance of the dry film, as mentioned above. Figure S21 displays that the obtained hydrogel can be readily cut into arbitrary structures, indicating high flexibility in shape and size tailoring. As a direct consequence of water swelling, the Zn(TFSI)_2_-post-treated PEDOT:PSS decreases the electrical conductivity from 892.4 S cm^–1^ for the dry film to 269.7 S cm^–1^ for the swollen hydrogel (Figure 4f), which is comparable to the highest electrical conductivity reported for hydrogels in the literature.^11,12,22^ The physical origin underlying such a conductivity reduction can be interpreted by the increase in the conductive inter-PEDOT domain distances during hydration.^67^ The conduction mechanism of the obtained hydrogel is electron-dominant, as revealed by the high-frequency inductive tail approaching the real axis of the Nyquist plot at lower frequencies (Figure 4g and Figure S22).^75,76^ Being highly conductive and flexible, the PEDOT:PSS hydrogel strip can undergo continuous bending, twisting, and stretching without causing any circuit disconnection, as reflected by the negligible brightness changes of the green light emitting diode (LED) bulb (Figure 4h). The high electrical conductivity of PEDOT:PSS hydrogel is also confirmed by an electrically self-healing test. Even during the cutting of hydrogel with a razor blade, the LED light driven by 2.8 V remains on due to the fast electrical recovery of the damaged area (Movie S3). For the application as a strain sensor, the conductive hydrogel is encapsulated by a poly(dimethylsiloxane) (PDMS) elastomer to avoid dehydration. The inset in Figure 4i shows the optical image of the as-fabricated strain sensor and the corresponding testing process. When subjected to continuous index finger bending at angles of 30°, 45°, and 55°, this strain sensor can output clear and reproducible signals, as evidenced by distinct electrical resistance changes (Figure 4i). The continuous bending process of the index finger is shown in Movie S4. With high flexibility, stretchability, electrical conductivity, mechanical stability, and fast electrical self-healing behavior, the obtained hydrogel could offer more possibilities for further exploration of wearable electronics and bioelectronics toward practical applications.

Furthermore, taking advantage of the instinctive hygroscopic nature of PSS moieties, we explore the humidity-responsive actuation using the Zn(TFSI)_2_-post-treated PEDOT:PSS as the single active material for the actuating device. As schematically illustrated in Figure S23, a simple custom-designed setup is used for controllable water vapor generation, thus creating a humidity gradient between the washcloth surface and air interface. Accordingly, the humidity in the ambient atmosphere and near the wet washcloth (containing ca. 46.8 wt % water) is measured to be ca. 27.9% and ca. 82.8%, respectively (Figure S24). Typical of water absorption/desorption-induced volume expansion/contraction, the moisture-sensitive actuator is capable of converting the humidity stimuli into the driving force for mechanical movement, such as bending.^10,26^ To evaluate such a responsive capability in a quantitative manner, single-layered actuators composed of rectangular PEDOT:PSS strips (*l* × *w*: 20 mm × 3 mm) of two different thicknesses are used to track the moisture-responsive bending angle as a function of time. When the actuator is placed on top of the washcloth, the instantaneously generated humidity gradient across the actuator propels the bending displacement. Due to the rapid absorption of water vapor, the thinner PEDOT:PSS active layer (thickness: 57.7 μm) exhibits a fast response within 1 s and achieves a maximum bending angle of 102° within 3 s (Figure 4j). More details about such an actuating behavior are recorded in a video (Movie S5). The bending angles of both strips are found to monotonically increase with time, while the time required for bending to the maximum angle increases from 3 s/108° for the thinner strip to 7 s/67° for the thicker strip (thickness: 218.8 μm) (Figures 4k and S25). Such a distinct discrepancy in actuating capability caused by thickness variations can be rationalized from the following two aspects: (i) As a result of less homogeneous water evaporation during the casting (Figure S26), the thicker strip inevitably generates more internal stress, thus deteriorating efficient actuation. (ii) Accompanied by the heavier weight, the thicker strip will compromise the rapid and highly efficient humidity response due to a gravity effect. A schematic representation of the working principle of such a single-component PEDOT:PSS-based actuator is shown in Figure 4l. In its original static state, the PEDOT:PSS active layer is in equilibrium with the ambient environment, with conducting-but-hydrophobic PEDOT-rich domains networks surrounded by hydrophilic-but-insulating PSS matrix. When in close proximity to the moist surface, the bottom interface of the actuator is endowed with a higher moisture content than the top surface due to the water absorption within the PSS matrix, which instantly induces a humidity gradient across the thickness direction.^77^ As a result of such an asymmetric swelling, a bending deformation away from the moist surface is triggered (Figure 4l and Movie S6). Upon humidity exposure, the inter-PEDOT domain spacings enlarge, while the PEDOT domain sizes remain almost unaffected since only the PSS moieties are water-sensitive, as revealed by our previous work.^67,68,75^ Such structural features play a beneficial role in inheriting the high conductivity of dry PEDOT:PSS to some degree, even during hydration. With passive PEDOT domains, the gradual water uptake in the PSS domains induces stresses in the active layer, which are minimized by the bending displacements.^10^ In view of its good conductivity and moisture-responsive actuation, such a Zn(TFSI)_2_-post-treated PEDOT:PSS actuator is further explored by functioning as a moisture-driven soft switch. As indicated by the light on and off state of a green LED in the circuit, the actuator strip reversibly establishes or breaks the circuit when an index finger approaches or moves away from the actuator surface (Movie S7). The rapid response to moisture evaporation from the human skin indicates the high humidity-responsive sensitivity of this single-layer actuator. Additionally, the Zn(TFSI)_2_-post-treated PEDOT:PSS-based actuator can be used in the field of bionic robots. Movie S8 and Figure S27 show the crawling motion of an inchworm-like robot triggered by the human finger. As the human finger approaches the inchworm-like robot slowly, the curvature of the robot gradually decreases. Correspondingly, the curvature progressively recovers to its initial state as the human finger moves far away. Initiated by the water gradient generated between the printing paper substrate and the human finger, such asymmetric film deformation would cooperate with film gravity and substrate friction to drive further film locomotion. Regarding humidity-responsive actuation, we also construct a biomimetic flower using the pentagram-shaped PEDOT:PSS film. As shown in the top panel of Figure S28, such a biomimetic PEDOT:PSS-based flower lies flat on a specific plane if no external stimulus is applied. When exposed to a humid atmosphere, the five petals of the PEDOT:PSS flower bend inward and envelope the screw within 5 s (bottom panel in Figure S28 and Movie S9), reminiscent of the closure of the leaves of Mimosa pudica when being touched. These results highlight the significant potential of highly conductive and moisture-responsive PEDOT:PSS films in smart applications of biosensors, bionic robots, and smart packaging.

## Conclusion

In summary, we have successfully developed a simple yet effective ionic salt post-treatment strategy for freestanding PEDOT:PSS films to achieve a combination of advantageous characteristics, including competitive electrical properties, high water stability, excellent mechanical flexibility, and fast moisture responsiveness. Among the choices of ionic salts, Zn(TFSI)_2_ yields the best electrical properties due to its highest capability in controlling the electrostatic assembly of PEDOT:PSS and corresponding chain conformation, oxidation levels, and microstructural characteristics. Accompanied by physical cross-linking via π–π stacking, continuous electrically percolated PEDOT network structures form and thus contribute to improved electrical and mechanical properties. With such a high cross-linking level, the hydrophobic PEDOT polymer chains, in turn, stabilize the dry PEDOT:PSS film against the occurrence of structural disintegration in water. Attributing to such integrated favorable properties, a single Zn(TFSI)_2_ post-treated PEDOT:PSS material enables further multifunctional applications in organic thermoelectric generators, highly conductive hydrogels, and moisture-responsive actuators. Our work presents a feasible strategy for overcoming multiple trade-off relations in PEDOT:PSS conducting polymer but also provides in-depth insights into the underlying improvement mechanisms. Furthermore, this work is anticipated to provide more guidelines for accessing PEDOT:PSS with more desirable properties for high-performance wearable devices, bioelectronics, and soft robotics, ultimately contributing to the development of a future multifunctional technology.

## Experimental Section

### Materials

Aqueous poly(3,4-ethylenedioxythiophene):poly(styrenesulfonate) (PEDOT:PSS) (Clevios PH1000) was purchased from H.C. Starck, Germany. The concentration of the PH1000 suspension was 1.1–1.3 wt% solids in water and had a PSS to PEDOT weight ratio of 2.5:1. 1-ethyl-3-methylimidazolium bis(trifluoromethylsulfonyl)imide (EMIMTFSI), lithium bis(trifluoromethanesulfonyl)imide (LiTFSI), and zinc di[bis(trifluoromethylsulfonyl)imide] (Zn(TFSI)_2_,) were all purchased from Sigma-Aldrich. Ethanol (≥99.8%), H_2_SO_4_ (98 wt%), and standard microscopy slides made of soda-lime glass were obtained from Carl Roth GmbH and Co. KG, Germany. p-doped silicon wafers with a thickness of 525 μm were provided by SiMat, Kaufering, Germany. Poly(dimethylsiloxane) (PDMS) (Sylgard 184) was bought from Dow Corning Corp., USA, with a mixture of base and cross-linker of 10:1 by mass. Sensitive disposable washcloths (composition: 65% viscose, 35% polyester, surface area: ca. 19 cm × 20.5 cm) were used as moist substrate. All solvents and chemical reagents in this study were used directly as received.

### Preparation of Pristine PEDOT:PSS Film

The PEDOT:PSS solutions were drop-casted onto acid cleaned and oxygen-plasma-treated glass substrates. All films were subsequently dried at 60 °C in ambient atmosphere.

### Preparation of Salt-Post-Treated PEDOT:PSS Film

PEDOT:PSS solution starts to gel even with the small addition of ionic salts as shown in Figure S7. Therefore, post-treatment of PEDOT:PSS films with ionic salt as the medium was used instead. Salt-post-treatment was performed by drop-casting aqueous EMIMTFSI or LiTFSI or Zn(TFSI)_2_ with different molar concentrations in water on the pristine PEDOT:PSS films, letting take effect for 24 h at room temperature, and then sufficiently rinsed five times in a DI water bath and dried at 60 °C in ambient atmosphere.

### Preparation and Encapsulation of PEDOT:PSS Hydrogel

Zn(TFSI)_2_-post-treated PEDOT:PSS film was soaked into DI water for 60 s. The surface of hydrogels was wiped before measurements. The PEDOT:PSS hydrogel was embedded between two PDMS layers with two ends connected to copper wires. PDMS was cured at room temperature and in sealed high-humidity conditions to avoid dehydration of the hydrogel.

### Thermoelectric Measurements and Characterization

Seebeck coefficients were measured using a home-built apparatus.^46^ As shown in Figure S29, the samples were placed between one hot copper block and one water-cooled copper block to create different temperature gradients. The temperature-dependent conductivity measurements were conducted using a home-built sealed setup with a filled N_2_ atmosphere such that the absence of water vapor was ensured. Before putting into this sealed chamber, the samples were thermally annealed to remove any water residuals. Thus, no hydration changes would occur within all PEDOT:PSS films during the temperature-dependent conductivity measurements. Sheet resistances were obtained with a four-point probe setup, by measuring on several different spots and averaging the values. All optical microscopy (OM) measurements were performed using an Axio Lab microscope (Carl Zeiss GmbH, Jena, Germany). Atomic Force Microscopy (AFM) images of the PEDOT:PSS films were acquired using an AFM instrument (Nanosurf, FlexAFM, Switzerland) in tapping mode. Contact potential differences were recorded in constant height mode via a Ti/Ir (5/20) coated Si cantilever tip. SEM measurements (N-vision 40 SEM, Zeiss) were carried out using secondary electron mode with an in-lens detector for material-sensitive measurements. Raman spectra were recorded using a 532 nm laser excitation on a Raman spectrometer (InVia Reflex, Renishaw). The AC impedance was measured with a potentiometer (WMP-300, Biologic, France) at 23 °C. Infrared spectra of samples were measured using a Fourier transform infrared spectrometer (FTIR, PerkinElmer Frontier) with an attenuated total reflectance sampling accessory. The ultraviolet–visible-near-infrared (UV–vis-NIR) absorption spectra of PEDOT:PSS films were acquired by a spectrophotometer (Lambda 35, PerkinElmer). The thicknesses of the PEDOT:PSS films were determined by a Bruker DektakXT surface profiler. The static GIWAXS/GISAXS measurements for PEDOT:PSS films were performed by an in-house instrument (GANESHA 300 XL SAXS SYSTEM by JJ X-ray Systems ApS) with the X-ray photon energy of 8.05 keV. The sample–detector distance (SDD) for GIWAXS and GISAXS was 96 mm and 1045 mm, respectively, and a Pilatus 300 K detector was used, having a pixel size of 172 μm × 172 μm. For cross-sectional SEM observations, the PEDOT:PSS hydrogel was frozen in a commercial refrigerator under −22 °C, followed by freeze-drying (Christ alpha 1–2 Lyophylle) under −50 °C and 0.0004 mbar for 48 h.

## Supporting Information Available

Modeling of GISAXS data, the self-made setup for humidity gradient generation and moisture-responsive quantification, thickness, optical micrographs, TE properties, AFM phase images, GIWAXS characterization, AC impedance, FTIR spectra, and cross-sectional SEM images of PEDOT:PSS films, digital photographs of PEDOT:PSS solutions and as-prepared pure and Zn(TFSI)_2_-post-treated PEDOT:PSS films, equivalent electrical circuit of a thermoelectric generator, demonstrations of the wearable TE device, moisture-responsive actuation of thick Zn(TFSI)_2_-post-treated PEDOT:PSS film (PDF)
Supporting movie S1: Demonstration of thermovoltage generation from hot water heat resource. (MP4)
Supporting movie S2: Zn(TFSI)2-post-treated PEDOT:PSS film soaked in water to prepare the hydrogel. (MP4)
Supporting movie S3: Electrically self-healing capability of the hydrogel after cutting. (MP4)
Supporting movie S4: Demonstration of the hydrogel strain sensor during finger bending. (MP4)
Supporting movie S5: Actuating behavior of PEDOT:PSS actuator driven by moisture. (MP4)
Supporting movie S6: Moisture-induced asymmetric swelling behavior of PEDOT:PSS actuator. (MP4)
Supporting movie S7: PEDOT:PSS actuator as a moisture-responsive switch. (MP4)
Supporting movie S8: Crawling motion of an inchworm-like robot triggered by a human finger. (MP4)
Supporting movie S9: The closing movements of a biomimetic flower when placed upon a moist substrate. (MP4)
