# Supplementary material for: Electrostatic Tailoring of Freestanding Polymeric Films for Multifunctional Thermoelectrics, Hydrogels, and Actuators
Source: ACS Nano. 2024 Dec 9;18(51):34829–41. doi: 10.1021/acsnano.4c12502 (PMC11673577; doi:10.1021/acsnano.4c12502)
Supplement: Supplementary file 1 — nn4c12502_si_001.pdf [file nn4c12502_si_001.pdf]

Supporting Information

Electrostatic Tailoring of Freestanding Polymeric Films for Multifunctional Thermoelectrics, Hydrogels, and Actuators

Suo Tu,[a‡] Ting Tian,[a‡*] Jinsheng Zhang,[a] Suzhe Liang,[a] Guangjiu Pan,[a] Xiaoxin Ma,[b] Liangzhen Liu,[b] Roland A. Fischer,[b] Peter Müller-Buschbaum[a,*]

[a]Technical University of Munich, TUM School of Natural Sciences, Department of Physics, Chair for Functional Materials, James-Franck-Str. 1, 85748 Garching, Germany

[b]Chair of Inorganic and Metal-Organic Chemistry, Department of Chemistry and Catalysis Research Center (CRC), TUM School of Natural Sciences, Technical University of Munich, 85748 Garching, Germany

[‡]These authors contributed equally

Corresponding Author

ting.tian@ph.tum.de (T.T.); muellerb@ph.tum.de (P.M.-B.)

Modeling of GISAXS data

The distorted wave Born approximation is used to model horizontal line cuts to acquire lateral structural information using the effective interface approximation[1]. The relationship between the differential cross-section and the solid angle can be expressed:

$$\frac{d\sigma}{d\Omega} = \frac{A\pi^2}{\lambda^4}(1-n^2)^2|T_i|^2\left|T_f\right|^2 P_{diff}(\vec{q}) \propto P_{diff}(\vec{q}) \qquad \text{Equation (S1)}$$

which depends on the size of the illuminated surface (A), the used wavelength (λ), the refractive index (n), and the Fresnel transmission functions of the incident (T_i) as well as scattered beams (T_f). $P_{diff}(\vec{q})$ denotes the diffuse scattering factor. Here, the Fresnel transmission functions only act as scaling factors because of the fixed incident and exit angles in the GISAXS measurements. As a result, the intensity is proportional to $P_{diff}(\vec{q})$. As is common in small-angle scattering, $P_{diff}(\vec{q})$ in relation to form factor $F(\vec{q})$ and structure factor $S(\vec{q})$ is determined as:

$$P_{diff}(\vec{q}) \propto N|F(\vec{q})|^2 S(\vec{q}) \qquad \text{Equation (S2)}$$

assuming N identical objects are arranged in a geometrical pattern, and $F(\vec{q})$ is the Fourier transform of the electron density distribution ρ_e. As a consequence, it is tightly bound to the shape and size of the scattering objects.

$$F(\vec{q}) = \int_V \rho_e\,(\vec{r})e^{2\pi i(\vec{q}\vec{r})}dV \qquad \text{Equation (S3)}$$

The local monodisperse approximation (LMA) is used in conjunction with a 1D paracrystal configuration of the scattering objects. The scattering objects' polydispersity is described with a Gaussian distribution function. In our modeling, a Gaussian distribution function describes the polydispersity of the scattering objects.

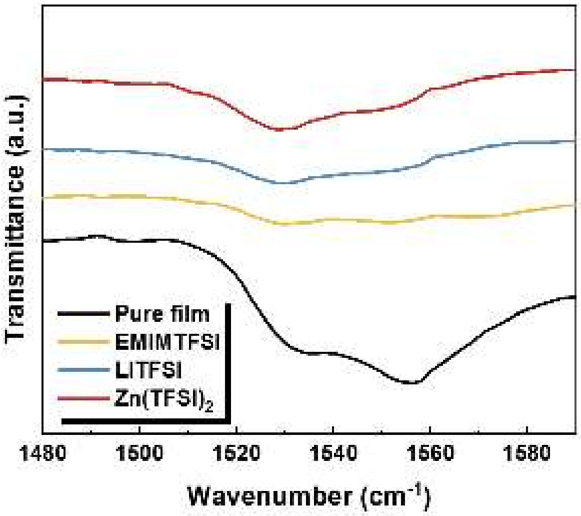

Figure S1. FTIR spectra of PEDOT:PSS films by various salt post-treatments within the range of 1480 to 1590 cm^{-1}.

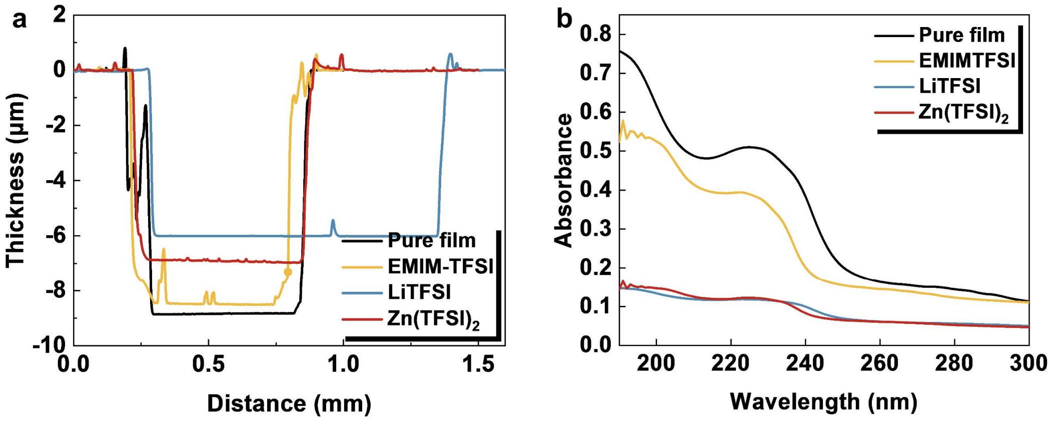

Figure S2. (a) Thickness determination of PEDOT:PSS films. The thickness loss after film post-treatment gives a clue that PSS is removed from the film. (b) UV absorption spectra of PEDOT:PSS films before and after the post-treatment. The two bands in the UV region reduced strongly after post-treatment and also indicate the removal of PSS moieties.[2]

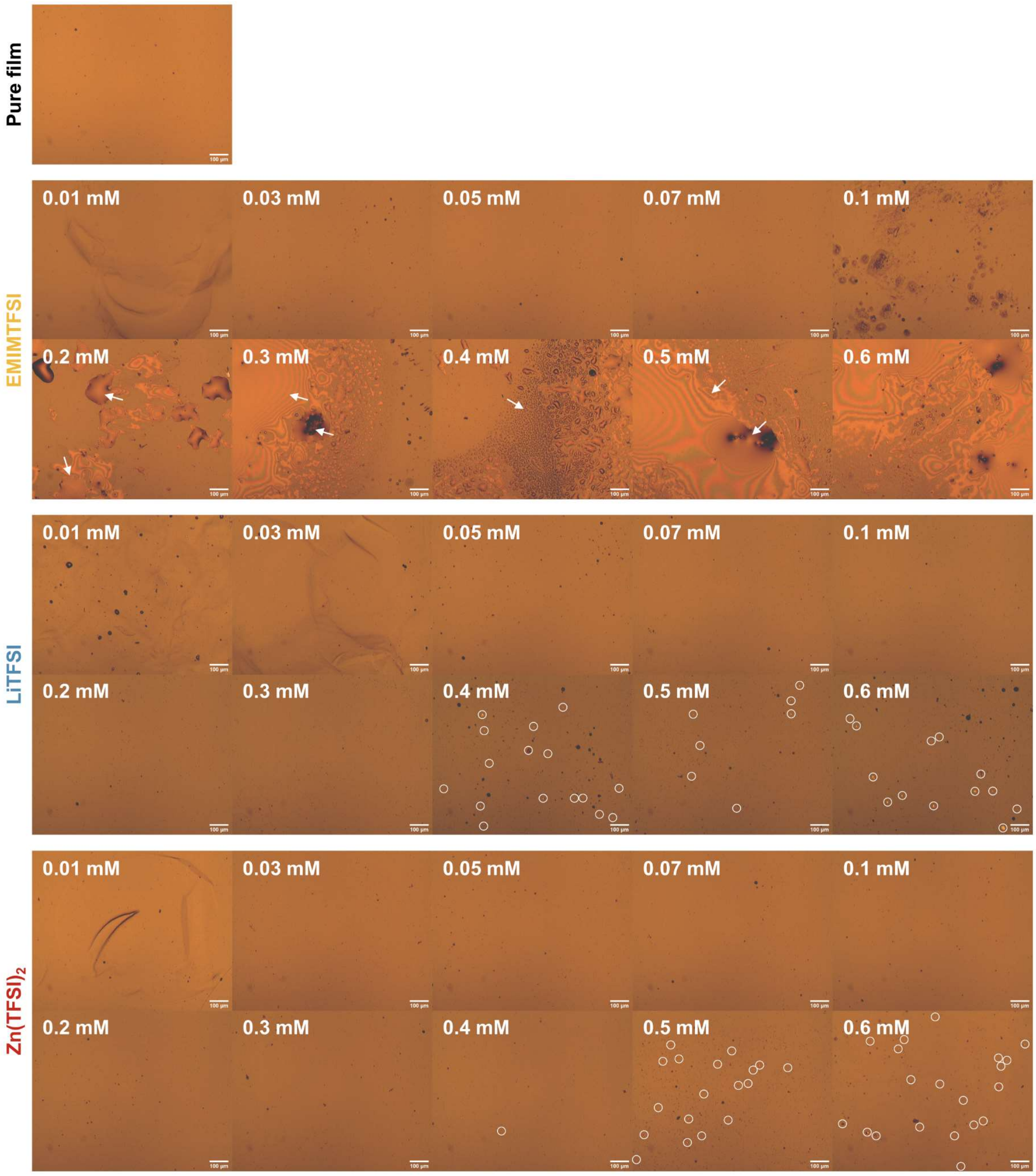

Figure S3. Optical micrographs of PEDOT:PSS films post-treated with three ionic salts at different molar ratios. White arrows and circles represent salt residues. The scale bar represents 100 μm.

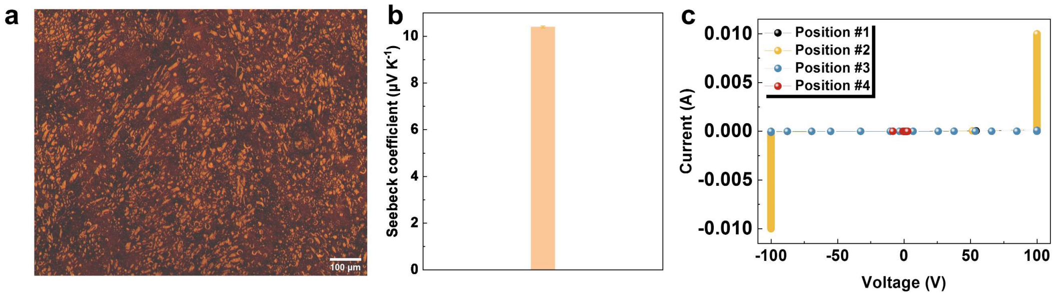

Figure S4. (a) Optical micrograph, (b) Seebeck coefficient and (c) sheet resistance of 0.4 mM $Zn(TFSI)_2$ post-treated PEDOT:PSS film without the rinsing process. The scale bar in Figure S4a represents 100 μm. As shown in Figure S4b and S4c, there is a drop in S and no detectable sheet resistance for the $Zn(TFSI)_2$ post-treated PEDOT:PSS film without the rinsing process, indicating the adverse effect of residual salt.

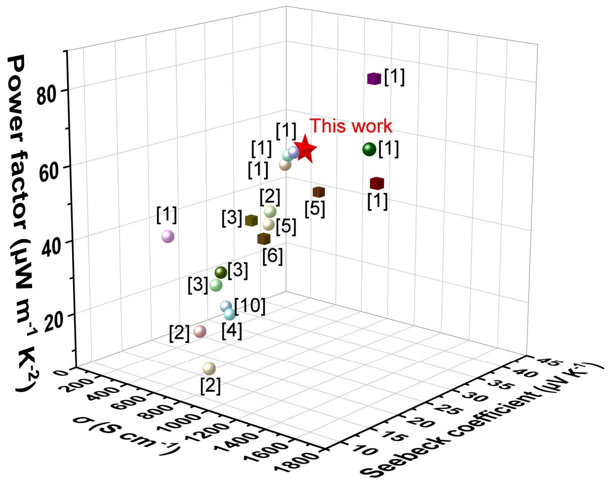

Figure S5. Comparison of the TE performance of salt-treated PEDOT:PSS films in the literature.[3-8]

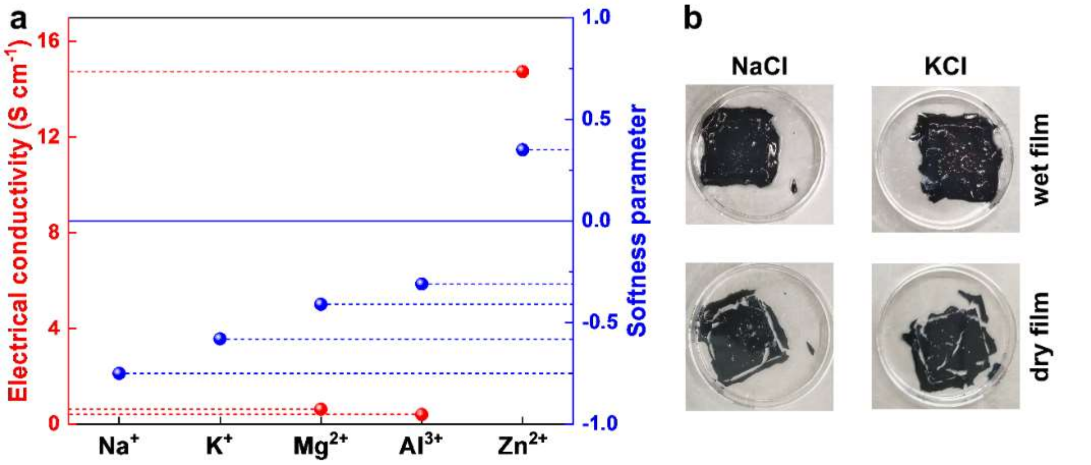

Figure S6. (a) Relationship between the softness parameter of cations of metal chlorides and the electrical conductivity of PEDOT:PSS films post-treated with the optimal salt concentration. (b) Digital photographs of 0.4 mM NaCl- or KCl-post-treated PEDOT:PSS films in wet and dry states. With more negative softness parameters, NaCl or KCl post-treatment leads to incomplete PEDOT:PSS films (as shown in Figure S6b) and thus, undetectable conductivities (as shown in Figure S6a).

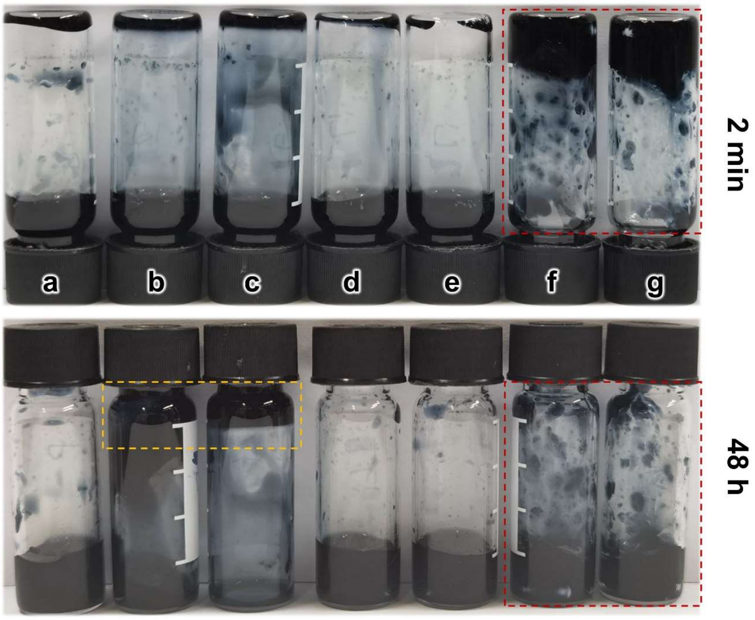

Figure S7. Digital photographs of PEDOT:PSS solutions: (a) without mixing and after mixing with (b) 0.01 mM EMIMTFSI; (c) 0.02 mM EMIMTFSI; (d) 0.01 mM LiTFSI; (e) 0.02 mM LiTFSI; (f) 0.01 mM $Zn(TFSI)_2$; (g) 0.02 mM $Zn(TFSI)_2$. The vial inversion test shows evident gel-like aggregation (indicated by dashed rectangles) upon the positive-softness-parameter EMIMTFSI or $Zn(TFSI)_2$ addition.

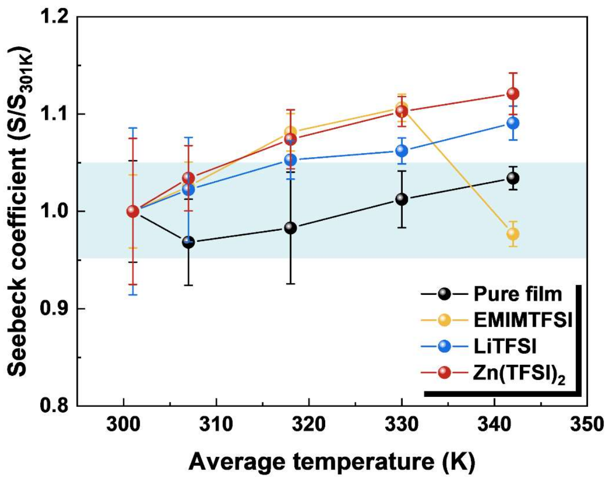

Figure S8. Temperature dependence of Seebeck coefficient of PEDOT:PSS films. The rectangle region represents the negligible variation (< 1 μV K^{-1}) within the resolution of the Seebeck tester.

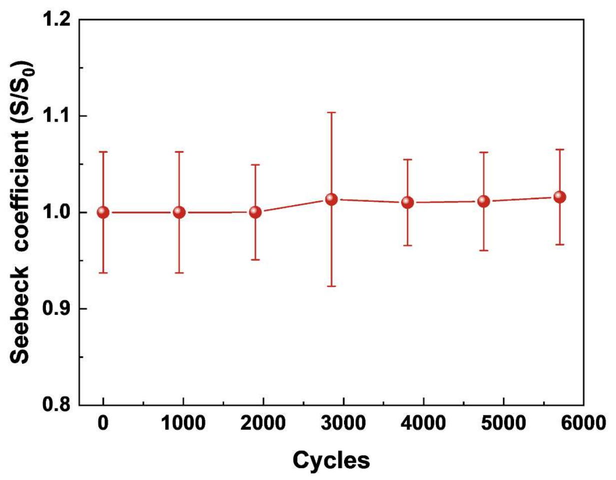

Figure S9. Seebeck coefficient variation of the Zn(TFSI)$_2$-post-treated PEDOT:PSS film under different mechanical bending cycles.

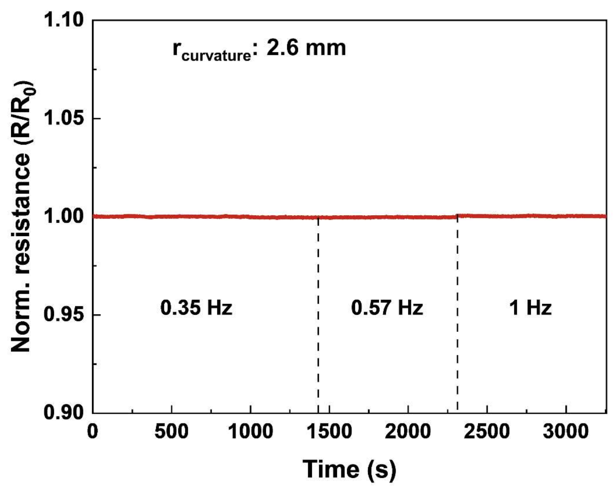

Figure S10. Normalized resistance of the Zn(TFSI)$_2$-post-treated PEDOT:PSS film bent for 950 cycles using different bending rates with a curvature radius of 2.6 mm.

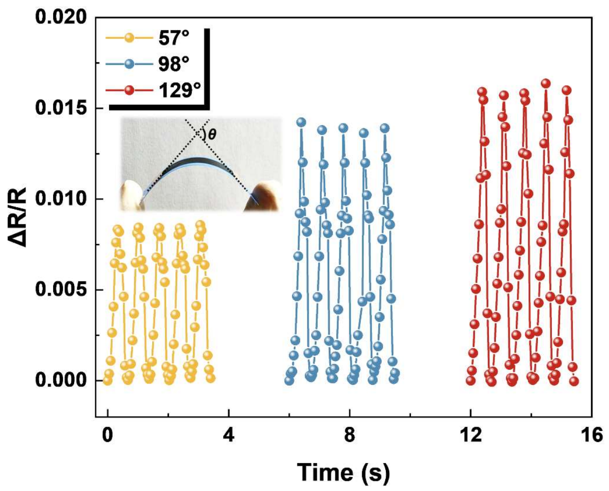

Figure S11. Resistance change *vs* bending angles of the $Zn(TFSI)_2$-post-treated PEDOT:PSS film. The inset is the digital photograph of resistance measurement while bending.

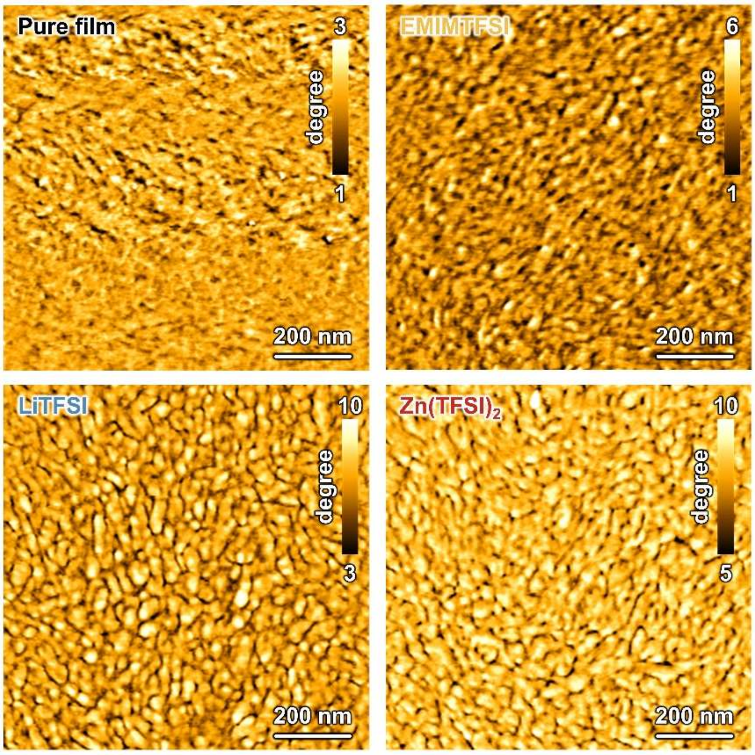

Figure S12. AFM phase images of PEDOT:PSS films.

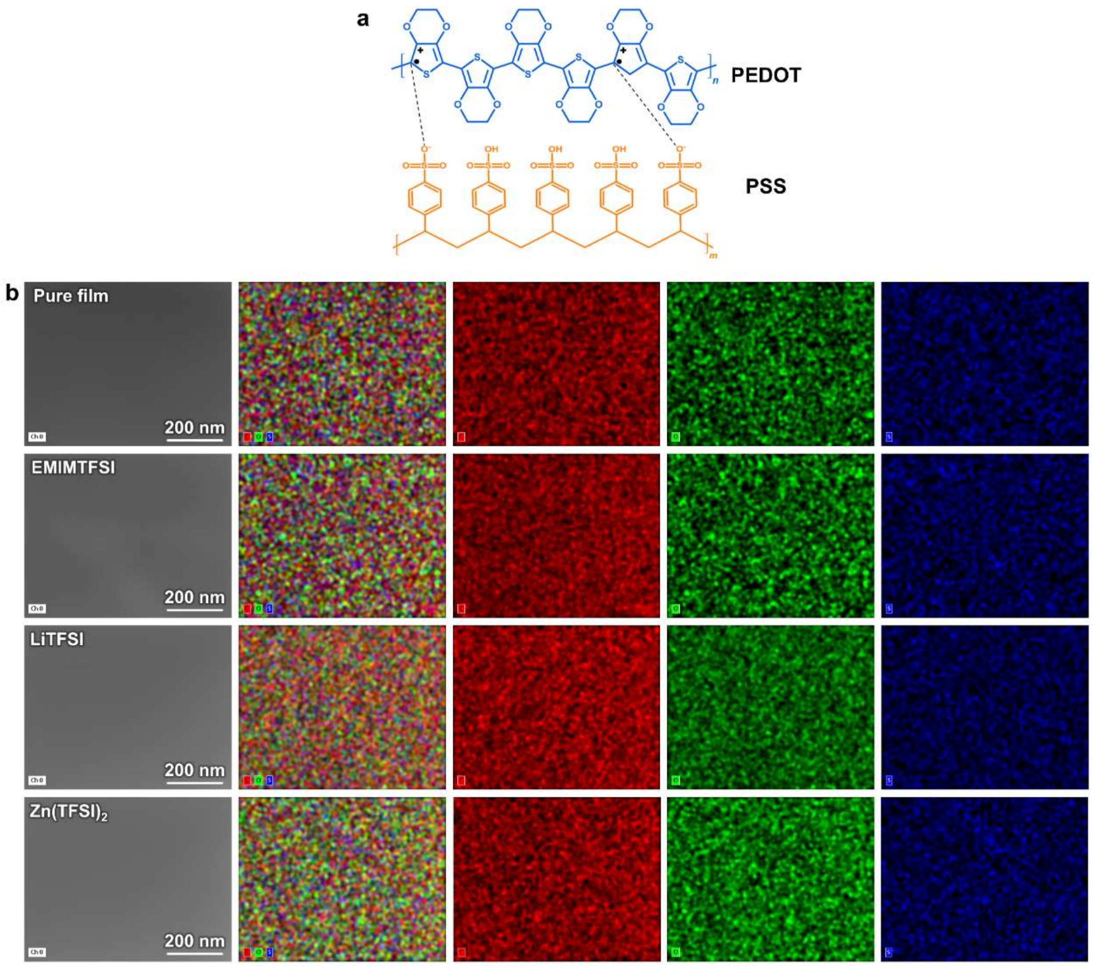

Figure S13. (a) Chemical structure of PEDOT:PSS. (b) SEM images of the PEDOT:PSS films upon various post-treatments and their corresponding elemental mapping images for C, O, and S. From left to right, SEM images, overlap of the EDS chemical elements distribution, C, O, and S mappings, respectively.

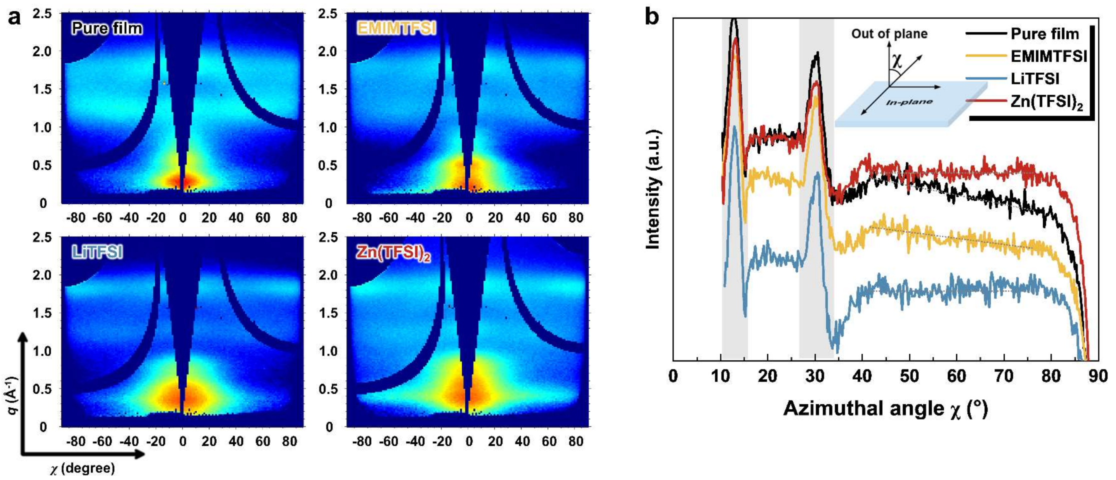

Figure S14. GIWAXS characterization of PEDOT:PSS films. (a) χ-reshaped 2D GIWAXS data and (b) the corresponding azimuthal tube cuts at the (010) Bragg peak of PEDOT of PEDOT:PSS films.

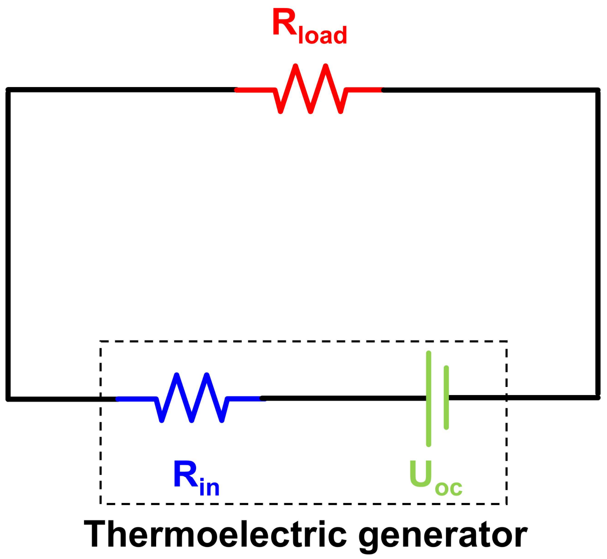

Figure S15. Equivalent electrical circuit of a thermoelectric generator. The current and voltage are measured using a variable load resistor.

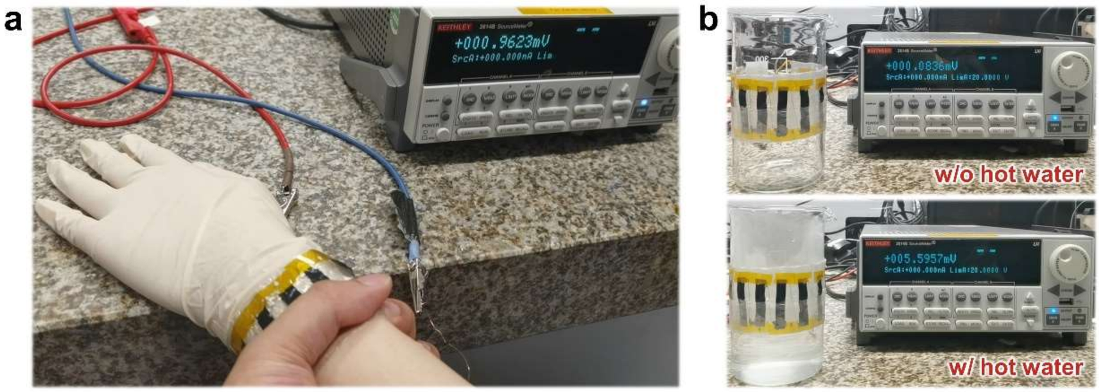

Figure S16. Simple demonstrations of the wearable TE device. It is worn on the curved surface of (a) the human wrist and (b) a beaker.

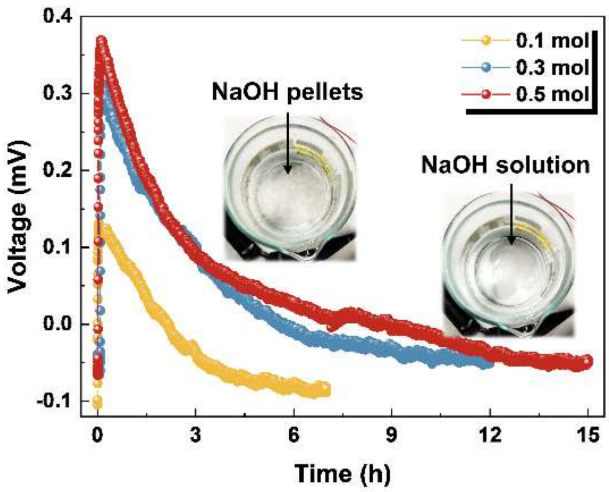

Figure S17. Generated voltage as a function of time for NaOH pellets with different molars dissolved in water. The inset shows photographs of NaOH pellets and NaOH solution.

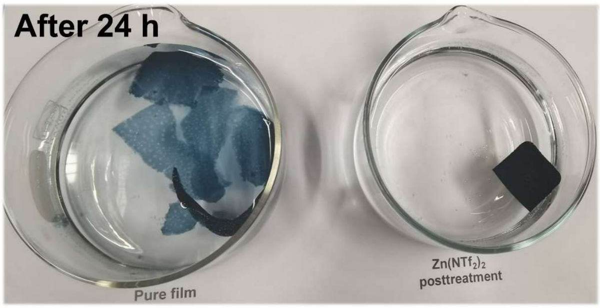

Figure S18. Digital photographs of as-prepared pure and Zn(TFSI)$_2$-post-treated PEDOT:PSS films after immersion in water for 24 h.

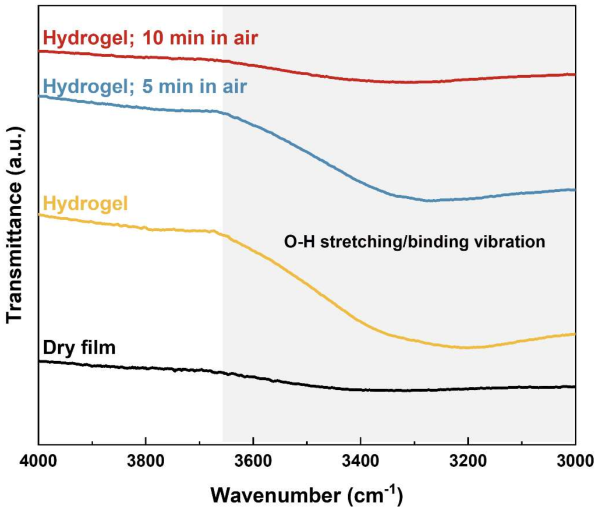

Figure S19. FTIR spectra of the Zn(TFSI)$_2$-post-treated PEDOT:PSS films before and after soaking in H_2O. The dehydration of the hydrogels occurs naturally in the air.

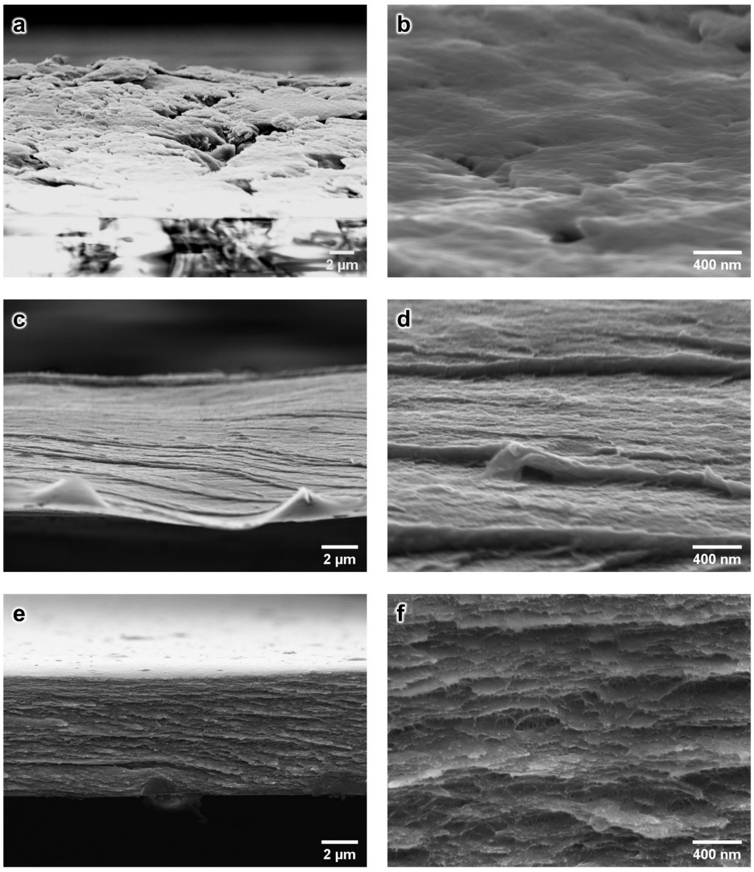

Figure S20. Cross-sectional SEM images: (a, b) pure film, (c, d) $Zn(TFSI)_2$-post-treated PEDOT:PSS film, and (e, f) $Zn(TFSI)_2$-post-treated PEDOT:PSS hydrogel after freeze-drying at different magnifications.

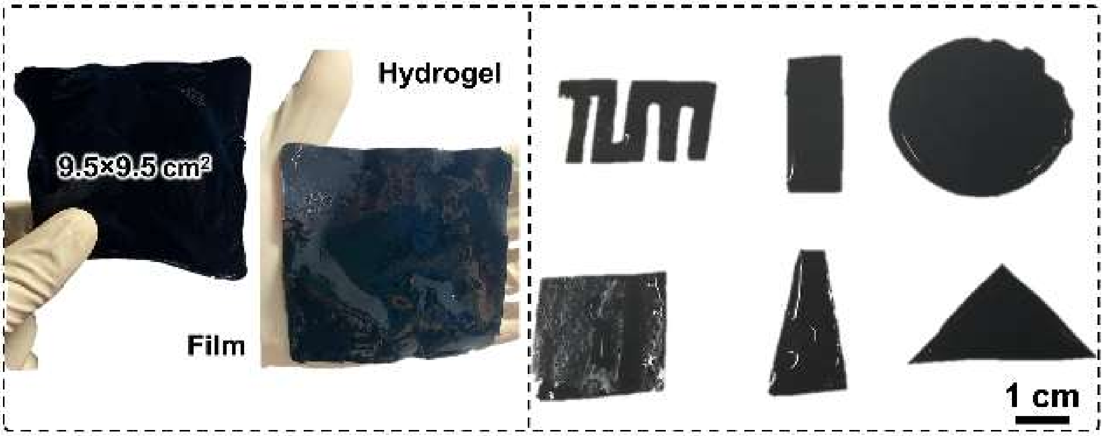

Figure S21. Digital photographs of $Zn(TFSI)_2$-post-treated PEDOT:PSS hydrogels with arbitrary shapes and dimensions.

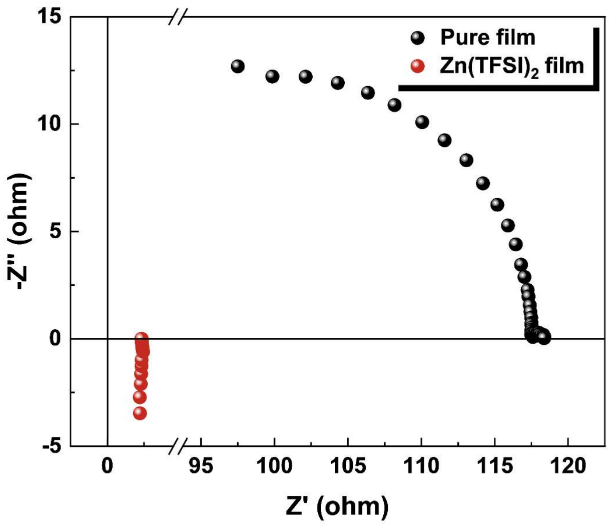

Figure S22. Nyquist plots of as-prepared pure film and $Zn(TFSI)_2$-post-treated PEDOT:PSS films at ambient conditions (RH<30%). The pure film shows one semicircle, typical of a parallel combination of a resistor and a constant phase element. The $Zn(TFSI)_2$-post-treatment eliminates the interface-induced capacitive effect, implying a purely electron-dominant conduction within the film.

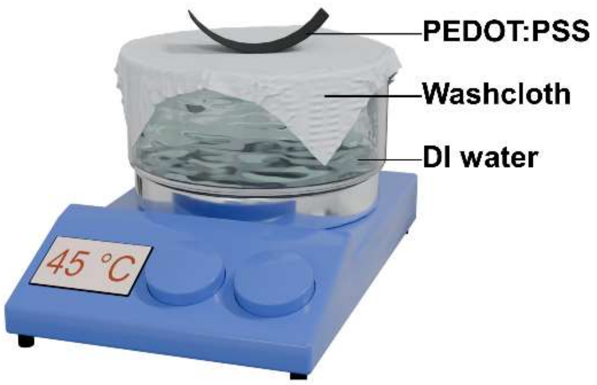

Figure S23. Schematic of the self-made setup for humidity gradient generation and moisture-responsive quantification.

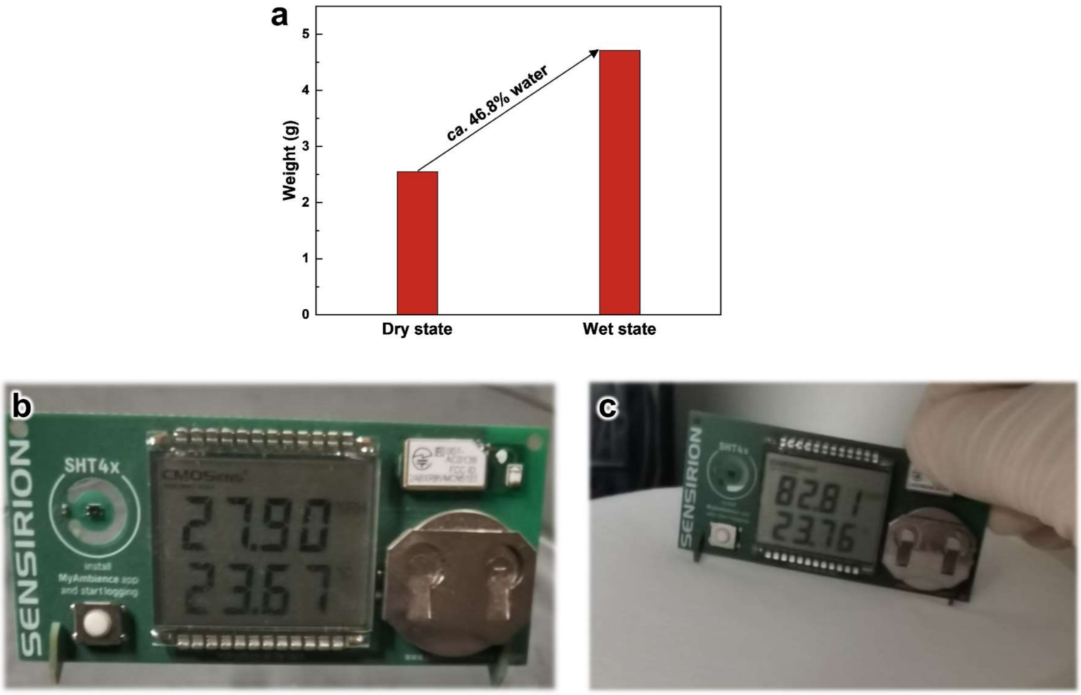

Figure S24. (a) Water weight test of the wet washcloth. (b) The ambient humidity and (c) humidity near the wet washcloth.

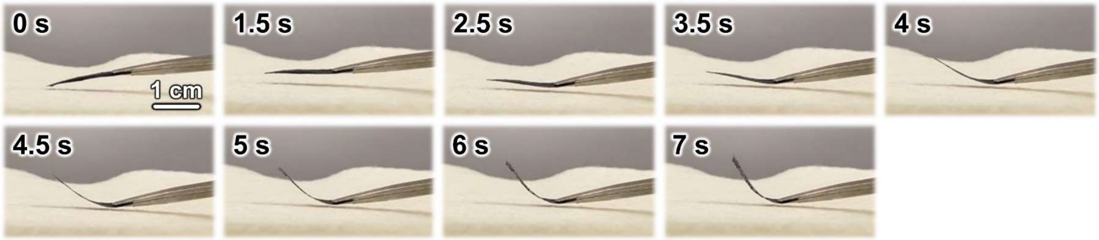

Figure S25. Digital photographs of the $Zn(TFSI)_2$-post-treated PEDOT:PSS strip ($l \times w \times h$: 20 mm × 3 mm × 218.8 μm) with time-dependent moisture response.

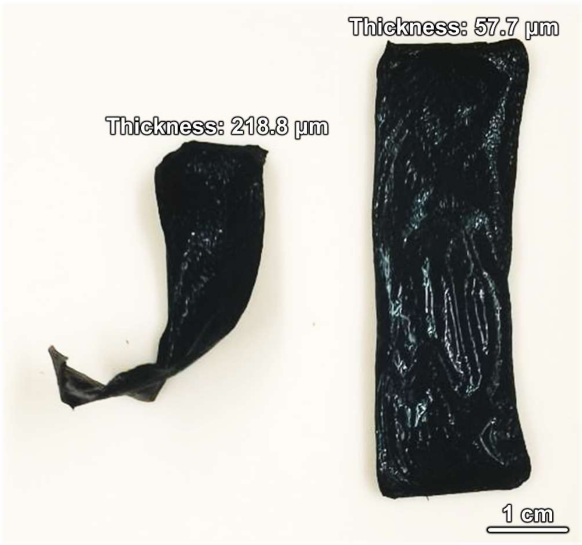

Figure S26. Digital photographs of $Zn(TFSI)_2$-post-treated PEDOT:PSS films.

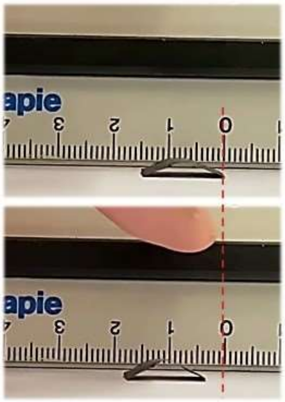

Figure S27. Crawling of an inchworm-like robot based on the $Zn(TFSI)_2$-post-treated PEDOT:PSS actuator being triggered by a human finger. Red dashed lines serve as guides to the eye.

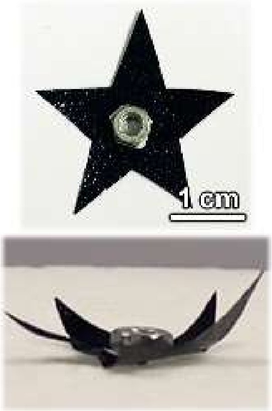

Figure S28. Moisture-responsive actuation of a starfish-shaped flower made of thick $Zn(TFSI)_2$-post-treated PEDOT:PSS film.

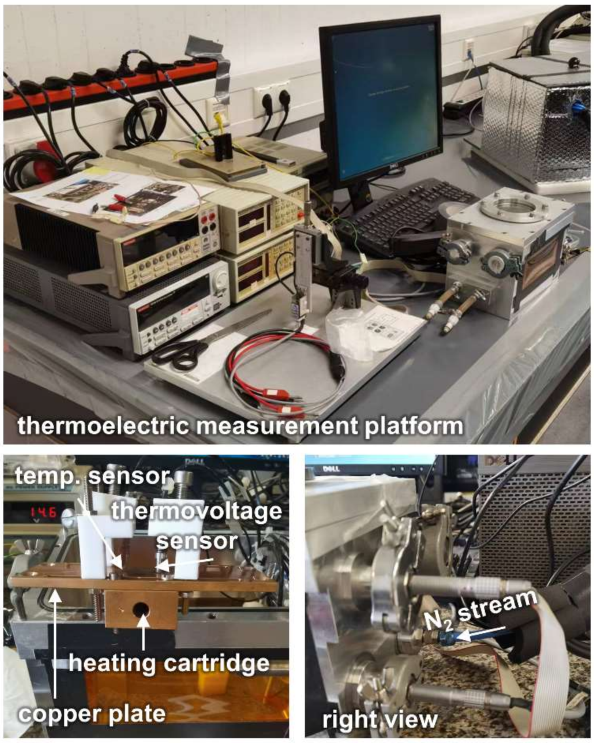

Figure S29. Digital photographs of home-built thermoelectric measurement platform.

Table S1 Comparison of the thermoelectric properties of drop-cast freestanding PEDOT:PSS/salts films with literature reported values.

Dopant	Treatment	σ (S cm^{-1})	S (μV K^{-1})	Reference
LiTFSI	salt addition	~418	-	*ACS Appl. Polym. Mater.* 3.1 (2020): 98-103.
BMIM BF_4	ionic liquid addition	~138	-	*Chem. Mater.* 19.9 (2007): 2147-2149.
BMIM OS	salt addition + salt solution rinsing	~288	-	*Sci. Adv.* 3.3 (2017): e1602076.
BIMBSO AT	salt addition + salt solution rinsing	~420	-	*Sci. Adv.* 3.3 (2017): e1602076.
LiTFSI	salt addition+salt solution rinsing	~608	-	*Sci. Adv.* 3.3 (2017): e1602076.
HMIM BF_4	salt addition+DMSO post-treatment	~804	~21	*J. Mater. Chem. A* 10.36 (2022): 18792-18802.
BMIM BF_4	salt addition+DMSO post-treatment	~679	~23	*J. Mater. Chem. A* 10.36 (2022): 18792-18802.
EMIM BF_4	salt addition+DMSO post-treatment	~533	~25	*J. Mater. Chem. A* 10.36 (2022): 18792-18802.
MMIM BF_4	salt addition+DMSO post-treatment	~434	~26	*J. Mater. Chem. A* 10.36 (2022): 18792-18802.
EMIM TCM	salt addition	~800	-	*Adv. Mater.* 34.12 (2022): 2109904.
EMIM ES	salt addition	~400	-	*Adv. Mater.* 34.12 (2022): 2109904.
EMIM Cl	salt addition	~200	-	*Adv. Mater.* 34.12 (2022): 2109904.
$NaClO_4$/p-FA	formic acid post-treatment +$NaClO_4$ post-treatment	~787	-	*Sci. Adv.* 8.47 (2022): eabq8160.

$NaClO_4$/p-EG/PEDOT:PSS	EG post-treatment +$NaClO_4$ post-treatment	~601	-	*Sci. Adv.* 8.47 (2022): eabq8160.
$NaClO_4$/a-EG/PEDOT:PSS	EG addition+$NaClO_4$ post-treatment	~538	-	*Sci. Adv.* 8.47 (2022): eabq8160.
$NaClO_4$/p-MSA/ PEDOT:PSS	methanesulfuric acid post-treatment +$NaClO_4$ post-treatment	~1006	-	*Sci. Adv.* 8.47 (2022): eabq8160.
$CuCl_2$/a-EG/PEDOT:PSS	EG addition+CuCl post-treatment	~565	-	*Sci. Adv.* 8.47 (2022): eabq8160.
NH_4Cl/a-EG/PEDOT:PSS	EG addition+NH_4Cl post-treatment	~367	-	*Sci. Adv.* 8.47 (2022): eabq8160.
NaCl/a-EG/PEDOT:PSS	G addition+NaCl post-treatment	~412		*Sci. Adv.* 8.47 (2022): eabq8160.
**$Zn(TFSI)_2$**	**salt post-treatment**	**~ 892**	**~26**	**This work**

Supporting movie S1: Demonstration of thermovoltage generation from hot water heat resource. (MP4)

Supporting movie S2: $Zn(TFSI)_2$-post-treated PEDOT:PSS film soaked in water to prepare the hydrogel. (MP4)

Supporting movie S3: Electrically self-healing capability of the hydrogel after cutting. (MP4)

Supporting movie S4: Demonstration of the hydrogel strain sensor during finger bending. (MP4)

Supporting movie S5: Actuating behavior of PEDOT:PSS actuator driven by moisture. (MP4)

Supporting movie S6: Moisture-induced asymmetric swelling behavior of PEDOT:PSS actuator. (MP4)

Supporting movie S7: PEDOT:PSS actuator as a moisture-responsive switch. (MP4)

Supporting movie S8: Crawling motion of an inchworm-like robot triggered by a human finger. (MP4)

Supporting movie S9: The closing movements of a biomimetic flower when placed upon a moist substrate.

Reference

1. Müller-Buschbaum, P., A basic introduction to grazing incidence small-angle X-ray scattering. In *Applications of Synchrotron Light to Scattering and Diffraction in Materials and Life Sciences*, Springer: 2009; pp 61-89.
2. Alemu, D.; Wei, H.-Y.; Ho, K.-C.; Chu, C.-W., Highly conductive PEDOT: PSS electrode by simple film treatment with methanol for ITO-free polymer solar cells. *Energy Environ. Sci.* **2012,** *5* (11), 9662-9671.
3. Luo, J.; Billep, D.; Waechtler, T.; Otto, T.; Toader, M.; Gordan, O.; Sheremet, E.; Martin, J.; Hietschold, M.; Zahn, D. R., Enhancement of the thermoelectric properties of PEDOT: PSS thin films by post-treatment. *J. Mater. Chem. A* **2013,** *1* (26), 7576-7583.
4. Atoyo, J.; Burton, M. R.; McGettrick, J.; Carnie, M. J., Enhanced electrical conductivity and Seebeck coefficient in PEDOT: PSS via a two-step ionic liquid and NaBH4 treatment for organic thermoelectrics. *Polymers* **2020,** *12* (3), 559.
5. Liu, C.; Xu, J.; Lu, B.; Yue, R.; Kong, F., Simultaneous increases in electrical conductivity and Seebeck coefficient of PEDOT: PSS films by adding ionic liquids into a polymer solution. *J. Electron. Mater.* **2012,** *41*, 639-645.
6. Saxena, N.; Pretzl, B.; Lamprecht, X.; Bießmann, L.; Yang, D.; Li, N.; Bilko, C.; Bernstorff, S.; Müller-Buschbaum, P., Ionic liquids as post-treatment agents for simultaneous improvement of Seebeck coefficient and electrical conductivity in PEDOT: PSS Films. *ACS Appl. Mater. Interfaces* **2019,** *11* (8), 8060-8071.
7. Mazaheripour, A.; Majumdar, S.; Hanemann-Rawlings, D.; Thomas, E. M.; McGuiness, C.; d'Alencon, L.; Chabinyc, M. L.; Segalman, R. A., Tailoring the seebeck coefficient of PEDOT: PSS by controlling ion stoichiometry in ionic liquid additives. *Chem. Mater.* **2018,** *30* (14), 4816-4822.
8. Jiang, K.; Hong, S.-H.; Tung, S.-H.; Liu, C.-L., Effects of cation size on thermoelectricity of PEDOT: PSS/ionic liquid hybrid films for wearable thermoelectric generator application. *J. Mater. Chem. A* **2022,** *10* (36), 18792-18802.
